# Elucidating gene expression adaptation of phylogenetically divergent coral holobionts under heat stress

**DOI:** 10.1038/s41467-021-25950-4

**Published:** 2021-09-30

**Authors:** Viridiana Avila-Magaña, Bishoy Kamel, Michael DeSalvo, Kelly Gómez-Campo, Susana Enríquez, Hiroaki Kitano, Rori V. Rohlfs, Roberto Iglesias-Prieto, Mónica Medina

**Affiliations:** 1 Biology Department, The Pennsylvania State University, University Park, PA USA; 2 Ecology and Evolutionary Biology Department, University of Colorado Boulder, Boulder, CO USA; 3 Center for Evolutionary and Theoretical Immunology, University of New Mexico, Albuquerque, NM USA; 4 School of Natural Sciences, University of California, Merced, CA USA; 5 Thermo Fisher Scientific, Carlsbad, CA USA; 6 Unidad Académica de Sistemas Arrecifales Puerto Morelos, ICMyL, Universidad Nacional Autónoma de México, Cancún, Mexico; 7 The Systems Biology Institute, Tokyo, Japan; 8 Okinawa Institute of Science and Technology, Okinawa, Japan; 9 Department of Biology, San Francisco State University, San Francisco, CA USA; 10 Present Address: US Department of Energy Joint Genome Institute, Lawrence Berkeley National Laboratory, Berkeley, CA USA

**Keywords:** Evolutionary biology, Transcriptomics, Marine biology

## Abstract

As coral reefs struggle to survive under climate change, it is crucial to know whether they have the capacity to withstand changing conditions, particularly increasing seawater temperatures. Thermal tolerance requires the integrative response of the different components of the coral holobiont (coral host, algal photosymbiont, and associated microbiome). Here, using a controlled thermal stress experiment across three divergent Caribbean coral species, we attempt to dissect holobiont member metatranscriptome responses from coral taxa with different sensitivities to heat stress and use phylogenetic ANOVA to study the evolution of gene expression adaptation. We show that coral response to heat stress is a complex trait derived from multiple interactions among holobiont members. We identify host and photosymbiont genes that exhibit lineage-specific expression level adaptation and uncover potential roles for bacterial associates in supplementing the metabolic needs of the coral-photosymbiont duo during heat stress. Our results stress the importance of integrative and comparative approaches across a wide range of species to better understand coral survival under the predicted rise in sea surface temperatures.

## Abstract (web summary)

As corals struggle to survive under climate change, it is crucial to know whether they can withstand increasing seawater temperatures. Using a controlled thermal stress experiment across three divergent coral holobionts, this study examines metatranscriptomic responses to heat stress corresponding to the coral host, photosymbionts and associated microbiota.

## Introduction

**Fig. 1:**
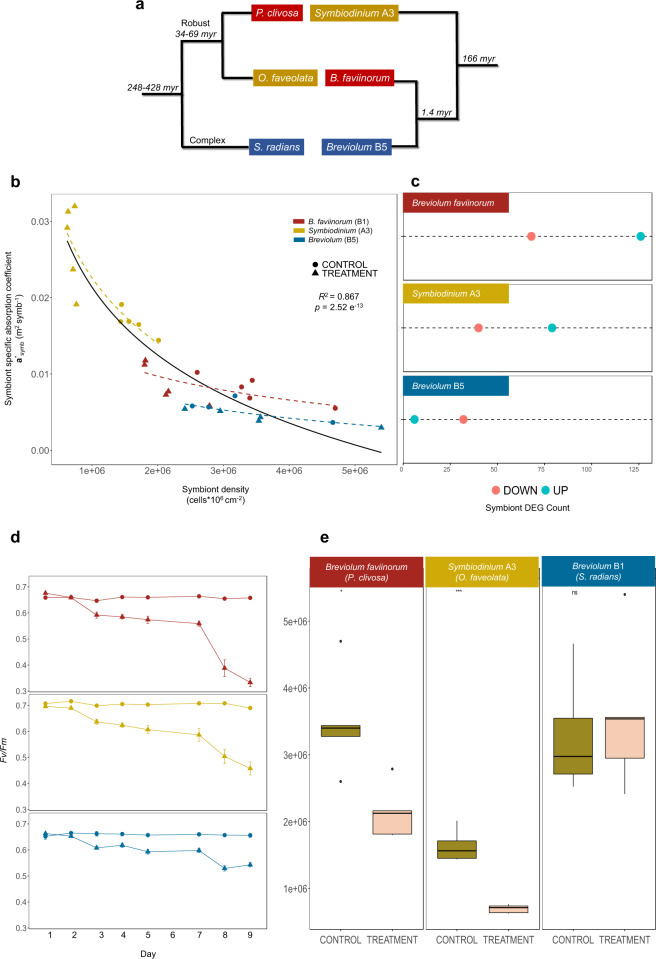
Comparative analysis of the Symbiodiniaceae physiological response across species. **a** Phylogeny representing the coral and associated photosymbiont species studied. A taxonomic color assignment is linked to host species throughout all figures. (Red: *P. clivosa*, Yellow: *O. faveolata*, Blue: *S. radians*). **b** Changes in the photosymbiont-specific absorption coefficient a*_sym_ as a function of photosymbiont density, this descriptor quantifies changes in the total effective area for light absorption experienced by each Symbiodiniaceae cell in hospite. **c** Number of DEGs after the heat treatment in each Symbiodiniaceae species (FDR < 0.001, – 2 > logFC > 2). The color assignment is linked to coral host (Red: *P. clivosa-B. faviinorum*, Yellow: *O. faveolata-Symbiodinium* A3, Blue: *S. radians-Breviolum* B5). Source data are provided as a Source Data file. **d** *Fv/Fm* (maximum quantum yield of PSII) measured during the nine days experiment between control (circle) (*n* = 5 *S. radians*, *n* = 5 *O. faveolata*, *n* = 6 *P. clivosa* biologically independent coral fragments) and treatment (triangles) samples (*n* = 5 *S. radians*, *n* = 5 *O. faveolata*, *n* = 6 *P. clivosa* biologically independent coral fragments). Data are presented as mean values + /– SEM. **e** Photosymbiont cell density in control (*n* = 4 *S. radians*, *n* = 5 *O. faveolata*, *n* = 5 *P. clivosa* biologically independent coral fragments) and treatment (*n* = 5 *S. radians*, *n* = 5 *O. faveolata*, *n* = 5 *P. clivosa* biologically independent coral fragments) samples. Asterisks depicts a statistical significance (*t*-test (two-sided), *B. faviinorum p* = 0.0081; *Symbiodinium* A3 *p* = 2.7e-05; ns *p* > 0.05; Supplementary Table 3). Boxplot whiskers show minima and maxima; centers indicate medians; box boundaries indicate the 25th and 75th percentiles.

A coral holobiont comprises the cnidarian host (Hexacorallia:Scleractinia) and its associated microbiome^1,2^ that functions within multiple interaction networks of emerging metabolic, physiological, and ecological capacities of all members^3^. Reef-building corals are characterized by a mutualistic symbiosis with unicellular algae (Dinophyceae:Symbiodiniaceae)^4^. These photosymbionts play a primary role in host physiology and nutritional exchange, enabling reef-building in oligotrophic environments^5^. Severe loss of the algal symbionts (i.e., bleaching) is associated with changes in light intensity, salinity, and episodes of elevated temperature^6^, with possible fatal consequences for the host. Cellular models of bleaching have been developed using a few host species with each study analyzing single taxa^7–11^ or without a phylogenetic framework in mind. Diverse ranges of thermal susceptibility across different coral holobionts may be partially explained by evolutionarily divergent differential gene expression that could be adaptive. A comparative approach is particularly germane for the dissection of adaptive potential by different holobiont partners under increasingly warmer oceans. By using a controlled thermal stress experiment across three divergent Caribbean coral holobionts (Fig. 1A), we aim to determine if there have been lineage-specific innovations or a conserved molecular response in corals and their algal photosymbionts, as well as whether all members (including bacterial communities) differentially contribute to holobiont robustness.

Here we show that (1) a core set of host and Symbiodiniaceae genes have diverged in their expression among three Caribbean coral species from the Robust (*Pseudodiploria clivosa*, *Orbicella faveolata*) and Complex (*Siderastrea radians*) clades respectively, and their photosymbionts (*Breviolum faviinorum*, *Symbiodinium* A3, and *Breviolum* B5 respectively); (2) microbiomes from heat resistant corals have an increased number of expressed metabolic capabilities when compared to heat-sensitive ones; and (3) the redundancy of key metabolic pathways from different holobiont members may confer robustness to the holobiont under heat stress.

## Results and Discussion

### Symbiodiniaceae physiology reveals a distinctive response to heat stress

We sought to establish whether the Symbiodiniaceae physiological response in hospite during heat stress was different between coral holobionts by measuring symbiont densities, reflectance at the peak of chlorophyll a absorption at 675 nm (R_675_) and the maximum photochemical efficiency of photosystem II (*Fv/Fm*). *A Fv/Fm* decrease quantifies the magnitude of the impact of light stress on the photosymbionts, expressed as a reduction in the maximum photochemical efficiency of the PSII pool due to photodamage accumulation (Supplementary Note 1). Symbiont density, specifically symbiont cell loss, is used here as an indicator to assess the heat-stress treatment impact. Estimated absorbance from reflectance determinations allowed the quantification of the effective light absorption cross-section of Symbiodiniaceae in hospite, a*_sym,_ and the magnitude of this variation for samples exposed to similar levels of heat and light stress. This optical trait is a proxy for the estimation of differences among coral species in the internal light environment of the symbionts in hospite, despite the coral exposure to a similar external light environment. By comparing the a*_sym_ and *Fv/Fm* it is possible to distinguish between the magnitude of the light pressure experienced by the symbionts in hospite and the level of photodamage accumulated. We found that *O. faveolata* photosymbionts (*Symbiodinium* A3), showed the largest increases in light pressure in hospite after heat stress, resulting in an important increment in a*_sym_ though they did not accumulate the largest photodamage (Fig. 1B). In contrast, *P. clivosa* symbionts (*Breviolum faviinorum*) under much lower variation in light pressure and despite the slight changes observed in the number of photosymbionts, exhibited the largest *Fv/Fm* drop after heat stress treatment (Fig. 1D).

Significant differences in a*_sym_ were observed between the three photosymbionts (ANOVA, *P* < 0.05; Fig. 1B). The low photosymbiont content of *O. faveolata* can explain the higher values estimated for *Symbiodinium* A3 *in hospite*, considering the negative non-linear association documented for both parameters^12^ and also here (Fig. 1B). However, significant differences in a*_sym_ were also observed between *Breviolum* spp. for corals harboring similar numbers of photosymbionts (Fig. 1B and Supplementary Tables S26-S28). This implies that *B. faviinorum* and *S. radians Breviolum* B5 photosymbionts were exposed *in hospite* to different light pressure levels. These differences are likely related to (1) intrinsic differences between photosymbiont species in their effective light absorption cross-section (a*_sym_); (2) the contribution of the different scattering properties of coral skeletons^13^ to modify a*_sym_ *in hospite* for each species; and (3) the presence of host pigments in *S. radians*^14^, which may have allowed reducing a*_sym_ *in hospite*, and level of light pressure and/or stress for the photosymbionts.

From these observations, it is remarkable that *Symbiodinium* A3 increased a*_sym_ during heat stress at higher risk of reaching the threshold of symbiosis disruption but counterbalancing high light stress when compared to the *B. faviinorum* (Fig. 1B). We interpret this as the *O. faveolata-Symbiodinium* A3 photosymbiont’s capacity to mount a robust response to high levels of light stress. Additionally, *O. faveolata‘*s ability to associate with multiple Symbiodiniaceae species (e.g., *D. trenchii*) during long periods of thermal stress could guarantee a faster photosymbiont tissue repopulation but at the cost of a lower calcification rate for the coral, as it has been documented by others^15^. In the case of *P. clivosa- B. faviinorum*, we observed a significant loss of photosymbionts during heat-stress, that resulted in the largest impact of light stress on *Fv/Fm*. This outcome, despite the number of remaining photosymbionts still high enough to moderate the increase in a*_sym_, suggests *B. faviinorum‘s* higher susceptibility *in hospite* to light stress. By contrast, in the *S. radians-Breviolum* B5 holobiont, all physiological parameters remained unaffected by the heat treatment (Fig. 1B, D, E and Supplementary Tables 2, 3, 9). Taken together all these results indicate that (1) *B. faviinorum* is a light-sensitive species in the mutualistic association with *P. clivosa*; (2) *Symbiodinium* A3 is a robust species to light stress when living in association with *O. faveolata*, whereas (3) *Breviolum* B5 living in association with *S. radians* results in a thermally resistant holobiont.

### Heat stress induces specific gene expression profiles in corals

**Fig. 2:**
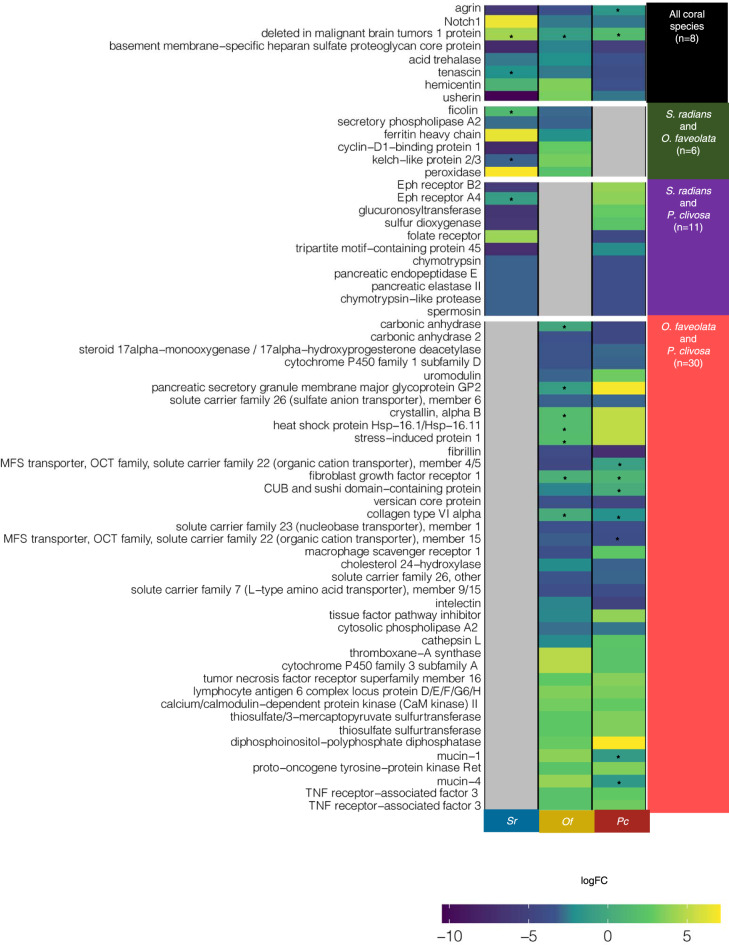
Significant Differential Expressed Genes (DEGs) across coral taxa. Each plot lines up DEGs according to their fold change in each species (FDR < 0.01). Asterisks denote variable expression from multiple transcripts with the same functional annotation (KO). Details on these genes with variable expression can be found in Tables S2, S4, S6, S8, S10, S12. Color assignment is linked to coral (Red: Pc *P. clivosa*, Yellow: Of *O. faveolata*, Blue: Sr *S. radians*).

The reference transcriptome assembly for each host resulted in 913,821, 731,432, and 685,205 transcripts for *O. faveolata*, *P. clivosa*, and *S. radians* respectively. While photosymbiont assemblies yielded 202,193, 184,910, and 173,968 transcripts for the corresponding associated Symbiodiniaceae (Supplementary Note 7 and Supplementary Fig. 1A). Orthology-based differential expression was performed by identifying orthologues using OrthoFinder^16^ and selecting a specific species transcriptional profile as the baseline for comparison of ortholog expression (Supplementary Fig. 2). We observed Differentially Expressed Genes (DEGs) with a similar profile pattern between the two-coral species from the same clade (Robust) and suborder (Faviina), *O. faveolata* and *P. clivosa*, when compared to the outgroup taxon *S. radians* (from the Complex clade, suborder Fungiina) (Fig. 2, Supplementary Fig. 2). Under heat stress, differential gene expression is more dynamic in the host (i.e., more genes mount an active transcriptional response), when compared to the number of significant DEGs in the photosymbiont (Supplementary Table 6).

We sought to determine if there was a functional transcriptional response to heat stress conserved across host taxa, beyond the strict orthology-based differential expression. By annotating the significant DEGs (*P* < 0.01) for each species with a KEGG ortholog (KO) (Supplementary Data 1), we retrieved genes coding for the same KO among host species. With this, we determine KEGG module/pathway enrichment of the upregulated and downregulated genes in the three coral hosts (*p* = 0.05, Supplementary Fig. 3A–C). In all three-coral species, the ECM-receptor interaction was an underexpressed KEGG category. This pathway includes proteins from the extracellular matrix, such as collagen types I, II, IV, and VI alpha, agrin, laminin, and heparan sulfate proteoglycan (perlecan), suggesting potentially a decrease in biomineralization. Collagen downregulation has been described in other coral species in response to heat stress/bleaching^9,11,17^. Its function is related to the extracellular matrix assembly, possibly with the cytoskeleton rearrangements, and the reductions in polyp and skeleton growth^18,19^ that take place during the stress before the disruption of the symbiotic relationship.

Only eight DEGs (KOs) were shared across all the host taxa, six genes were shared between *S. radians* and *O. faveolata*, 11 genes were shared among *S. radians* and *P. clivosa*, and 39 genes shared between the two closely related coral species, *O. faveolata* and *P. clivosa* (Fig. 2). From the eight host core DEGs, four genes shared the same expression profile (i.e., all are underexpressed): agrin, basement membrane-specific heparan sulfate proteoglycan core protein, tenascin, and acid trehalase, which is consistent with the previous KEGG enrichment results. Acid trehalase (K22078) has a conserved gene expression profile across host taxa. This gene codifies for an enzyme that breaks the carbohydrate trehalose, the accumulation of which has been suggested to play a role as an osmoprotectant in the development of symbiotic nitrogen-fixing root nodules^20^. During heat stress, this sugar-degrading enzyme may be downregulated in the coral hosts due to a compromised Symbiodiniaceae trehalose biosynthesis and/or the host preventing trehalose degradation to promote its accumulation as osmoprotectant against oxidative stress.

From this group of eight shared significantly expressed genes (KOs) across host species, there is one gene with variable inter and intraspecific variation, deleted in malignant brain tumors 1 (*dmbt1*). Each host species has several transcripts coding for dmbt1 differentially expressed (Fig. 2 and Supplementary Data 1). *dmbt1* has been postulated in regulating host-microbe interactions since it is at the interface between defense mechanisms and the homeostasis of cell proliferation^21^. Furthermore, *dmbt1* differential expression has been documented in *O. faveolata* upon lipopolysaccharide exposure^22^. In the coral *Acropora millepora*, *dmbt1* variable expression has been reported from corals with varying susceptibility to bacterial exposure. Corals with low-mortality and control fragments upregulate this gene, suggesting a significant role in the animal-microbe symbioses maintenance^23^. During the heat stress, we found two out of three *dmbt1* transcripts, highly expressed (logFC ~ 8) in *S. radians*, highlighting the relevance of the regulation of associated bacterial communities during heat stress.

Gene ontology enrichment analysis across taxa was employed to get a general representation of the biological processes differentially regulated during heat stress (Supplementary Data 3). Aminoglycan catabolism, keratan sulfate catabolic process, and sphingolipid metabolism were downregulated in all host species (Supplementary Data 3). Sphingolipid metabolism has been also documented as a part of a rheostat involved in the cnidarian heat stress response^24^. Consistent with the DEG orthology analysis, a large number of enriched GO terms is conserved in the closely related species, *O. faveolata* and *P. clivosa* when compared to *S. radians*. Diverse metabolite transport was down-regulated in both Faviina species (amino acid, sulfur amino acid, sodium ion, organic cation, L-cystine, bicarbonate, inorganic anion, coenzyme transport). Likewise, L-ascorbic acid and vitamin B6 metabolism, ammonia assimilation, and signal transduction involved in the cellular response to ammonium ion were down-regulated in heat-stressed corals (Supplementary Data 3). In contrast with the downregulation of Faviina metabolite transport, the thermal tolerant species*, S. radians*, upregulated genes involved in modified amino acid, inorganic anion transmembrane transport. Different gene transporters encoding carbonic anhydrase, belonging to the bicarbonate transport, have been identified as cnidarian endosymbiosis-related genes^25–27^. During heat stress, it is possible that the endosymbiosis disruption affects the nutrient, and metabolite transport across the coral host and its photosymbionts.

Differences in GO enriched terms among these species revealed an upregulation of genes related to DNA replication in *O. faveolata*, whilst *P. clivosa* displayed an opposite response. DNA conformational change, and repair, were upregulated in *O. faveolata*, as well as in *S. radians*, the heat susceptible coral, *P. clivosa* display a down-regulated response (Supplementary Data 3). Oxidative stress has been implicated as a key component in the cnidarian bleaching cascade triggered by heat stress^28^. The cellular response to oxidative stress, cell–cell adhesion, and cellular redox homeostasis was downregulated in the *S. radians* when compared with *P. clivosa*, where those GO terms were upregulated during heat stress. Reactive oxygen species response, endoplasmic reticulum to Golgi vesicle-mediated transport, protein folding in the endoplasmic reticulum, apoptotic signaling pathway, cellular amino acid, and short chain fatty acid catabolism, cellular response to glucose starvation, and monosaccharide biosynthetic process were up-regulated in both Faviina suborder coral species (Supplementary Data 3). The GO terms enriched in our dataset intersects with multiple other studies reporting similar biological processes in cnidarian studies investigating thermal stress and bleaching^8,11,17,28–30^.

GO enrichment analysis revealed a series of conserved and contrasting responses across the three corals investigated, highlighting the fact that while ortholog level expression might not be as conserved, overall functional conservation during heat stress can still be detected between distinct species, alluding to the fact that coral species might have adapted to utilize different genes to eventually achieve the same biological functions elicited during thermal stress. (See “Supplementary Note 2” for further dissection of host response).

### Symbiodiniaceae photosynthesis gene expression profile is key to heat stress resistance

**Fig. 3:**
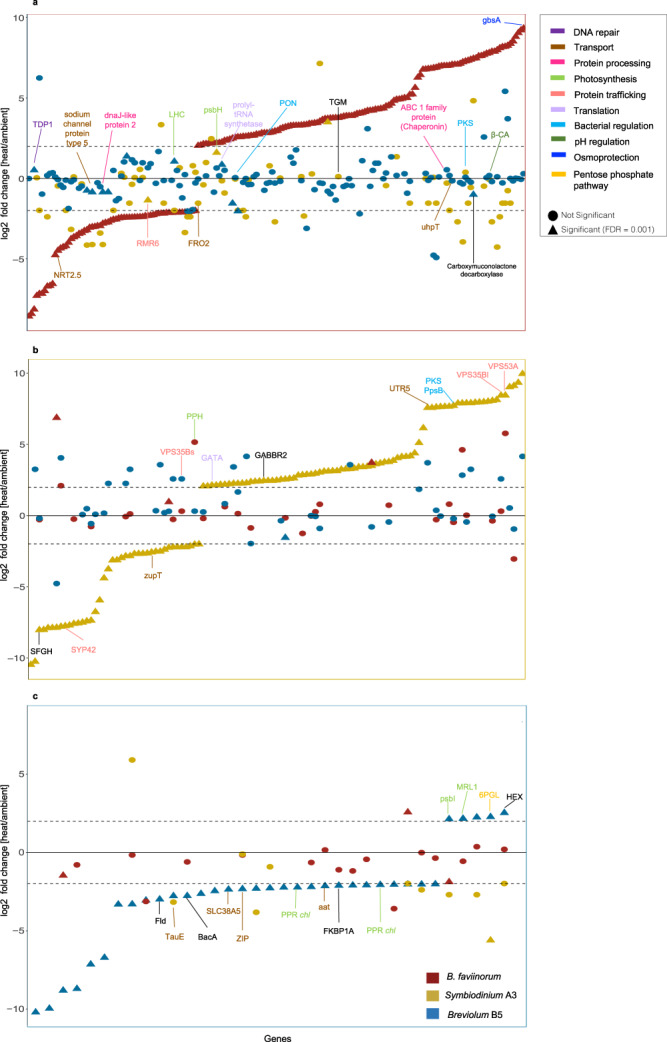
Fold change of Symbiodiniaceae ortholog expression during heat stress. *B. faviinorum* (**a**), *Symbiodinium* A3 (**b**), and *Breviolum* B5 (**c**) as a baseline and comparing the ortholog genes across the two Symbiodiniaceae species respectively (–2 > logFC > 2, FDR < 0.001). Each plot lines up DEGs according to their fold change in each species. Each plot also includes fold change for the homologs in other species. Color assignment is linked to coral host (Red: *P. clivosa,-B. faviinorum*, Yellow: *O. faveolata-Symbiodinium* A3, Blue: *S. radians-Breviolum* B5). Source data are provided as a Source data file.

Differential gene expression analyses in the photosymbiont partners revealed a pattern where in hospite *Breviolum* B5 photosymbionts displayed the smaller number of DEGs, *Symbiodinium* A3 exhibited an intermediate profile whereas *B. faviinorum* had the highest gene number at both flexible and strict FDR (*P* < 0.01; *P* < 0.001, respectively) (Fig. 3 and Supplementary Table 6). The transcriptional response mounted by Symbiodiniaceae partners is higher in the heat-stressed *P. clivosa* holobiont, when compared to its photochemical efficiency (Fig. 1D, B and Supplementary Table 6). In contrast, the heat resistant species *S. radians* exhibited fewer DEGs revealing a consistent association between symbiont photophysiology *in hospite* and the transcriptional profile displayed as a heat stress response in the photosymbiont partner.

Multiple cellular and metabolic genes involved in transport, metal homeostasis, and photosynthesis (Fig. 3 and Supplementary Data 1) were differentially expressed in the three Symbiodiniaceae species, although many transcripts remain unknown due to a lack of homology to annotated sequences. In most cases, the expression among orthologues was divergent across the three photosymbionts (Fig. 3 and Supplementary Data 1). The determination of significant DEG at a flexible and strict FDR (<0.001 and <0.01) did not uncover conserved gene expression profiles among photosymbiont taxa. Only one gene was conserved between the thermo-susceptible *B. faviinorum* and *Symbiodinium* A3, deoxyribodipyrimidine photo-lyase, a coding gene involved in the repair of UV radiation-induced DNA damage^31^.

When determining genes enriched in any GO category, a similar lack of conserved response across photosymbiont taxa is observed. Only one enriched GO, sodium ion transmembrane transport, is conserved in all three photosymbionts (Supplementary Data 3). However, this category is upregulated in *Breviolum* B5 and downregulated in the photosymbionts establishing symbioses with the Faviina suborder (*B. faviinorum* and *Symbiodinium* A3). Regardless of the photosymbiont phylogenetic relatedness, a higher number of GO categories were enriched and conserved in magnitude of expression among the Faviina associated photosymbiont species. Cation and metal ion transport were downregulated, and the regulation of RNA splicing and mRNA processing was upregulated (Supplementary Data 3).

At the gene expression level, we found multiple photosynthesis species-specific DEGs in response to increasing temperature. Photosynthesis impairment during heat stress severely compromises symbioses. High levels of PSII photoinactivation reduces photosynthesis rates, a phenomenon known as photoinhibition^32^. During this phase, the D1 protein is compromised due to the limitation of protein repair to cope with the high photodamage rates^33,34^. Although we did not observe a significant differential expression of D1 *psbA*; *psbH*, a gene involved in the PSII formation and necessary for photoprotection against oxidative damage, was overexpressed significantly in *B. faviinorum* (logFC = 2) and *Symbiodinium* A3 (logFC = 1.8), the two photosymbionts exposed *in hospite* to the highest light pressure (i.e., a*_sym_) and photodamage (i.e., drop in *Fv/Fm*). PsbH promotes the accelerated breakdown of photodamaged D1 protein, which is key to prevent the rapid accumulation of the degraded protein and subsequent photoinactivation. *psbH* expression profile reveals a photosymbiont response to increasing light stress *in hospite* during the heat stress treatment. This photosymbiont’s preemption to alleviate heat stress is in fact the expected algal response to light stress. If the duration of an elevated temperature episode is prolonged, D1 degradation and the repair rate will be exceeded by the amount of accumulated damaged PSII reaction centers, leading to photosynthesis inhibition and eventually to coral bleaching^35^. In contrast, *Breviolum* B5 employs a different strategy by overexpressing another PSII core complex gene, *psbI* (logFC = 2) which is downregulated in the other Symbiodinaceae species. *psbI* is associated with the D1/D2 heterodimer and plays a role in the assembly and stabilization of PSII dimers^36^. This response of the thermotolerant *Breviolum* B5 relies on the PSII stability rather than on its degradation, while *B. faviinorum* induced the genes involved in a degradation mechanism, highlighting the fact that while members of the same genus, these two photosymbionts employ quite different strategies to contend with photodamage and present very contrasting responses to heat stress.

Pentatricopeptide repeat-containing protein *mrl1* gene is overexpressed in the thermotolerant *Breviolum* B5. It has been shown that MRL1 regulates and stabilizes the RuBisCO transcript (*rbcL*), preventing its degradation^37^. This result, in conjunction with the overexpression of *psbI*, is consistent with the low levels of light pressure shown by *Breviolum* B5 *in hospite*, which allows this coral holobiont to cope with higher temperatures without compromising algal photosynthetic capacity. Indeed, *Breviolum* B5 is capable not only of transcribing genes involved in PSII core complex stabilization but also of synthesizing significant amounts of transcripts involved in chlorophyll biosynthesis, another indication of the low levels of light stress experienced *in hospite* during the heat treatment.

*Symbiodinium* A3 (*O. faveolata)* prevents the chlorophyll breakdown by under expressing pheophytinase (logFC = −2), whereas *B. faviinorum* tends to overexpress this gene (logFC = 5.15) and in B5, it is not differentially expressed. *Symbiodinium* A3 cell density is drastically reduced by the heat treatment (Fig. 1D). The inhibition of chlorophyll breakdown and the sustained PSII repair rate reflect an intermediate ability of the remaining *in hospite* cells to sustain photosynthesis under heat stress, contrary to the more sensitive *B. faviinorum*. Further evidence described in the following sections supports *Breviolum* B5 as a thermotolerant photosymbiont. (See “Supplementary Note 3” for further dissection of photosymbiont response).

### Baseline expression adaptation among coral and photosymbiont species changes during heat stress

By considering the evolutionary gene expression profiles along different coral lineages, we tested for evidence of selection and the adaptive potential in these species by using the expression variance and evolution (EVE) model, also known as Phylogenetic ANOVA^38^. This approach identifies candidate genes based on expression divergence in a phylogeny where samples are subject to ambient conditions. We developed an extension of the model, EVE-R (EVE-Response) to assess genes with divergent expression in ambient conditions between taxa, and change in expression upon a stimulus (i.e., heat stress) at least in one of the species. This set of genes displays a signature of expression adaptation and has expression levels sensitive to heat stress.

**Fig. 4:**
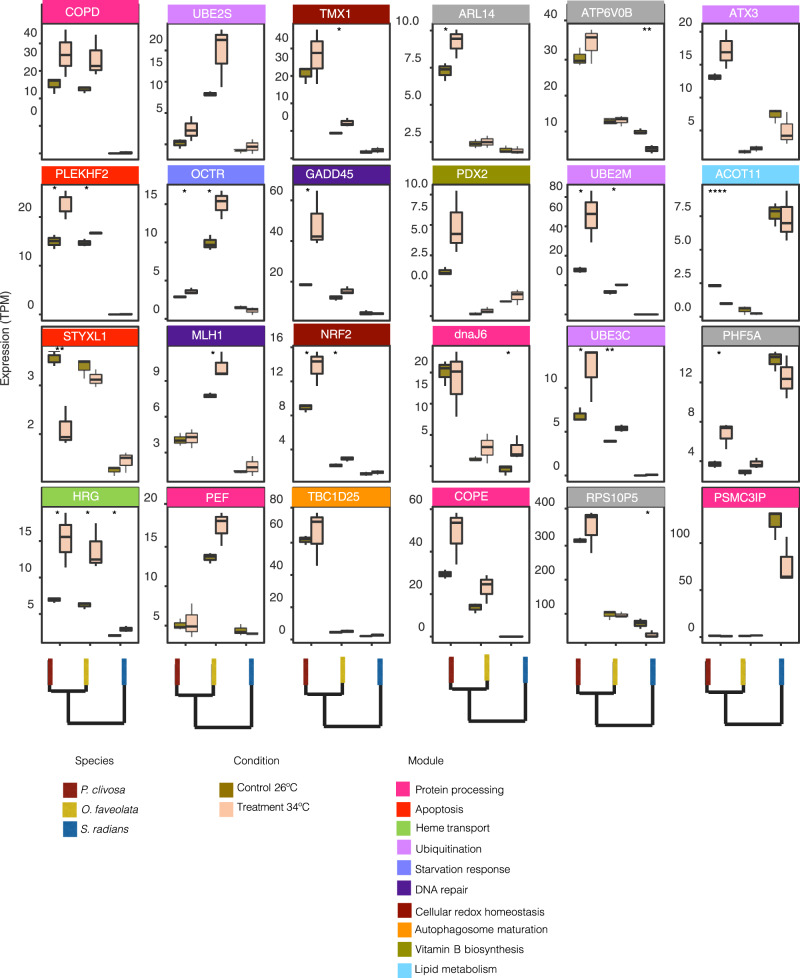
Coral host expression profiles for the genes identified by EVE-R. These genes have a divergent expression in ambient conditions, as well as a differential expression between ambient conditions (*n* = 3 *S. radians*, *n* = 3 *O. faveolata*, *n* = 3 *P. clivosa* biologically independent coral fragments) and after heat stress treatment (*n* = 3 *S. radians*, *n* = 3 *O. faveolata*, *n* = 3 *P. clivosa* biologically independent coral fragments). Boxplot whiskers show minima and maxima; centers indicate medians; box boundaries indicate the 25th and 75th percentiles. (Two-sided *t*-test *p* < 0.05; *p ≤ 0.05; **p ≤ 0.01; ***p ≤ 0.001; ****p ≤ 0.0001. Specific *p*-values for each comparison are provided in Supplementary Data 2). Source data are provided as a Source data file.

**Fig. 5:**
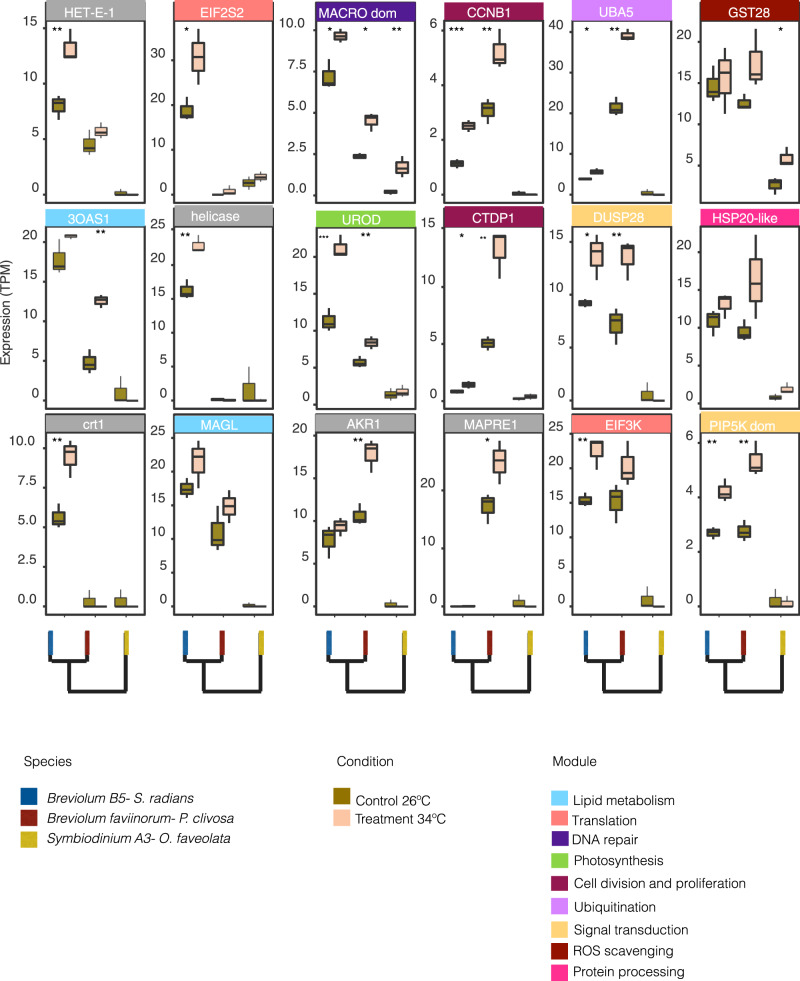
Symbiodiniaceae expression profiles for the genes identified by EVE-R. These genes have expression divergence in ambient conditions, as well as a differential expression between ambient (*n* = 3 *S. radians*, *n* = 3 *O. faveolata*, *n* = 3 *P. clivosa* biologically independent coral fragments) and heat stress (*n* = 3 *S. radians*, *n* = 3 *O. faveolata*, *n* = 3 *P. clivosa* biologically independent coral fragments) treatments. Boxplot whiskers show minima and maxima; centers indicate medians; box boundaries indicate the 25th and 75th percentiles. (Two-sided *t*-test *p* < 0.05; *p ≤ 0.05; **p ≤ 0.01; ***p ≤ 0.001; ****p ≤ 0.0001. Specific *p-*values for each comparison are provided in Supplementary Data 2). Source data are provided as a Source data file.

EVE-R found 67 candidates for divergent expression genes in the hosts and 39 in the photosymbionts. When gene expression levels for these potential adaptive lineage-specific genes changed in the basal expression for control samples *vs*. treated samples, we determined they were thermally induced. Divergent expression in ambient conditions, for genes involved in heat stress response, included 24 host and 18 photosymbiont genes (Figs. 4–5, respectively and Supplementary Data 2). No GO terms were significantly enriched (FDR 10%) for either the host or the photosymbiont. In general, host genes comprised protein processing, ER to Golgi vesicle-mediated transport, ubiquitination, lipid metabolism, metabolism of cofactors and vitamins, cell redox homeostasis, transport, autophagosome maturation, mRNA splicing, DNA repair, apoptosis, and presumably the carbon concentration mechanism.

A key candidate for expression level adaptation is the V-type protein ATPase 21 kDa subunit (ATP6V0B) (EVE divergence test *p* = 0.035), which is part of an ATPase complex acidifying the symbiosome^39^. This ATPase constitutes a key element in the association, as a carbon concentration mechanism that favors the protonation of HCO_3_ to form CO_2_ in the symbiosome to ensure a flux of CO_2_ towards RuBisCO to maintain its carboxylation activity and the synthesis of photosynthates that eventually are translocated to the coral host^40^. The inhibition of this proton pump blocks oxygen production, thus presumably may promote Symbiodiniaceae photosynthesis^39^. This mechanism could allow the host to exert active control on the photosymbiont physiological output in addition to other resource limitations (i.e., nitrogen). Under heat stress, however, carbon transport from the host may be restricted affecting algal photosynthesis. Corals such as *P. clivosa* susceptible to heat stress due to the high levels of light stress and photosynthesis inhibition experienced by the photosymbionts *in hospite*, may favor the CO_2_ supply for the Symbiodiniaceae, elevating mRNA levels of ATPase, in a host attempt to stimulate photosynthesis or photosynthates translocation. In *S. radians*, coupled host-algal translocation and photosynthesis likely remain unaffected supporting the low levels of stress experienced by this species under the experimental treatment.

Our differential expression analyses of metabolic transcripts show evidence for a loss of photochemical efficiency and an increase in photodamage in *P. clivosa-B. faviinorum*, impairing photosynthetic support in this system. When this ATPase (*atp6v0b*) expression is compared to *S. radians* we inferred a robust interaction between the thermotolerant host and its photosymbiont. We hypothesize that during a complex ROS feedback between the *S. radians* coral host and its *Breviolum* B5 photosymbiont, the host may be able to fine-tune the ROS production by slowing down the rate of photosynthesis. This coordinated regulation hints at a potential adaptive mechanism in heat resistant species by (1) allowing the photosymbiont to adjust the rate of photosystem repair, and (2) by decreasing ROS production in response to elevated temperature, mitigating the downstream events that lead to coral bleaching.

Our EVE-R model reveals that *gst-mu* (*gst28*/glutathione-S-transferase) has a lineage conserved expression in the genus *Breviolum* where both species expressed it at similar levels in ambient samples (EVE divergence test *p* = 0.032). This suggests a frontloaded expression response (i.e., elevated transcriptional baseline levels in response to heat stimulus previously documented in *Acropora hyacinthus* corals^17^) to the ROS generated during host melanin production^41,42^. *Breviolum* B5 photosymbionts express GST28 at similar levels in both control and treated samples, whereas *B. faviinorum gst28* expression in *P. clivosa* slightly increased upon heat stress (Fig. 5). Coral host melanin synthesis generates ROS and nitrogen intermediate compounds that have the potential to inhibit pathogens but can also cause self-damage^43^. Corals synthesize the antioxidants catalase, peroxidase and superoxide dismutase to alleviate oxidative stress^43,44^. Melanin levels are higher in *S. radians* whilst the more closely related corals (*P. clivosa, O. faveolata*) produce more prophenoloxidase^45^. Host melanin in *S. radians* seems to be actively produced, therefore the expression level adaptation for GST remains constitutively active in its photosymbiont. Additional evidence supporting a late-induction of melanin production after heat stress is overexpression of the melanocyte-stimulating hormone receptor in *P. clivosa* (logFC =  2.0) and underexpression in *S. radians* (logFC = − 3.12).

The rate of repair of the photosynthetic machinery is fundamental to understanding the disruption of coral-Symbiodiniaceae symbiosis. During the heat treatment stress and under normal conditions we observed that the uroporphyrinogen decarboxylase (*urod*) gene is highly expressed in *Breviolum* B5 in contrast to the photosymbionts of *P. clivosa* and *O. faveolata*. (Fig. 5) (EVE divergence test *p* = 0.023). This decarboxylase is involved in the formation of the precursor heme (iron) and chlorophyll through tetrapyrrole biosynthesis. The heme group is essential for the synthesis of porphyrins, specifically chlorophyll *chl.* a and *c2*. We did not perform any pigment analysis in this study, limiting our capacity to interpret this variation. The low levels of light stress experienced by *Breviolum* B5 *in hospite*, and measured as low accumulation of photodamage, may explain the maintenance of chlorophyll biosynthesis in the *S. radians-Breviolum* B5 association.

### Intrinsic expression adaptation in response to heat stress among coral and photosymbiont species

We developed a second EVE method variant to test directly for genes with divergent expression response to the environmental stimulus, EVEReSt (EVE in Response to Stimulus). Contrary to EVE-R we detected genes that are not divergent in ambient conditions but are responsive to heat stress. This method accounted for the intrinsic relative response to elevated temperatures by subtracting the treatment expression values from the control values in each species and testing for expression divergence with EVE. After running the EVEReSt model, the divergent expression gene candidates were considered adaptive genes for the species to contend with thermal perturbation.

A total of 63 host genes and 76 Symbiodiniaceae genes were considered divergent with a 0.05 *p*-value cutoff (Supplementary Data 2). Functional enrichment for this dataset was performed, however, no GO terms were significantly enriched (FDR 10%) for the host nor the photosymbiont. In general, the biological processes ; these genes span a range of essential cellular and metabolic pathways (e.g., starvation response, ever, Golgi pH regulation, iron transport, beta-oxidation, apoptosis, ubiquitination, phagosome maturation during heat stress).

We observed a transcriptional specific-species response with increasing expression of genes involved in DNA repair, protein processing, and degradation pathways on the thermosensitive *B. faviinorum* (Supplementary Data 2). The most divergent gene between the thermotolerant species and the susceptible species despite their phylogeny is the integral light-harvesting complex *acpPC*, a central gene in the symbiont photoacclimatory response to changes in light pressure. *acpPC* expression in the thermotolerant *Breviolum* B5 is higher when compared to *B. faviinorum* and *Symbiodinium* A3. This is consistent with photosynthesis genes overexpressed in *Breviolum* B5 (Fig. 3). The ability to actively synthesize the *acpPC* light-harvesting complex and *UROD* in *Breviolum* B5 reflects the absence of light stress during the exposure of *S. radians* to heat stress and more likely the reduction of the internal light environment of *Breviolum* B5 associated with the maintenance or even increased in photosymbiont number for some samples (Fig. 1B, D–E). This robust response to heat stress is determined by the ability of *S. radians* to maintain or even enhance the number of photosymbionts under stress. Whether this response is conferred by the host or by *Breviolum* B5, or both, remains unclear.

The EVEReSt method uncovered distinct gene expression profiles for the gene kynureninase in both host and photosymbionts highlighting the evolutionary importance of this enzyme in holobiont symbiosis (Supplementary Data 2). Kynureninase is similarly expressed in *P. clivosa* and its respective photosymbiont *B. faviinorum* but not expressed in either *S. radians* and *Breviolum* B5 or *O. faveolata* and its partner *Symbiodinium* A3. Kynureninase is an enzyme catalyzing the kynurenine (KYN) conversion to anthranilate and L-alanine. This enzyme is part of the KYN pathway involved in tryptophan degradation. In mice, KYN metabolite levels in induced gut dysbiosis decreased while Trp increased^46^. After inoculation of gut microbes, levels are restored^46,47^. Similarly, episodes of elevated temperature stimulate coral microbiome dysbiosis^48^, illustrating the importance of microbiome regulation. We observed a slight decrease and a distribution shift in the bacterial diversity of *P. clivosa* (Supplementary Fig. 4F) together with the increasing kynureninase expression. The enzyme kynureninase in *S. radians* is downregulated in both partners. This suggests that Coral-Symbiodinaceae KYN and Trp metabolism may play a role in the holobiont crosstalk with their microbiota and heat stress may contribute to its disruption.

Consistent with a bacterial diversity decrease in *P. clivosa* and *O. faveolata* the differential expression analysis revealed the overexpression of polyketide synthase and a potential microbial quorum sensing quencher gene (paraoxonase/arylesterase 1) in *P. clivosa* and its photosymbiont (*B. faviinorum*) as well as in *Symbiodinium* A3, the *O. faveolata* photosymbiont (Fig. 3A, B and Supplementary Data 2). The resulting protein products could be interfering with the microbiota’s survival and growth.

### Comparative quantitative metabolic fingerprint reveals a distinct role of holobiont members upon temperature increase

**Fig. 6:**
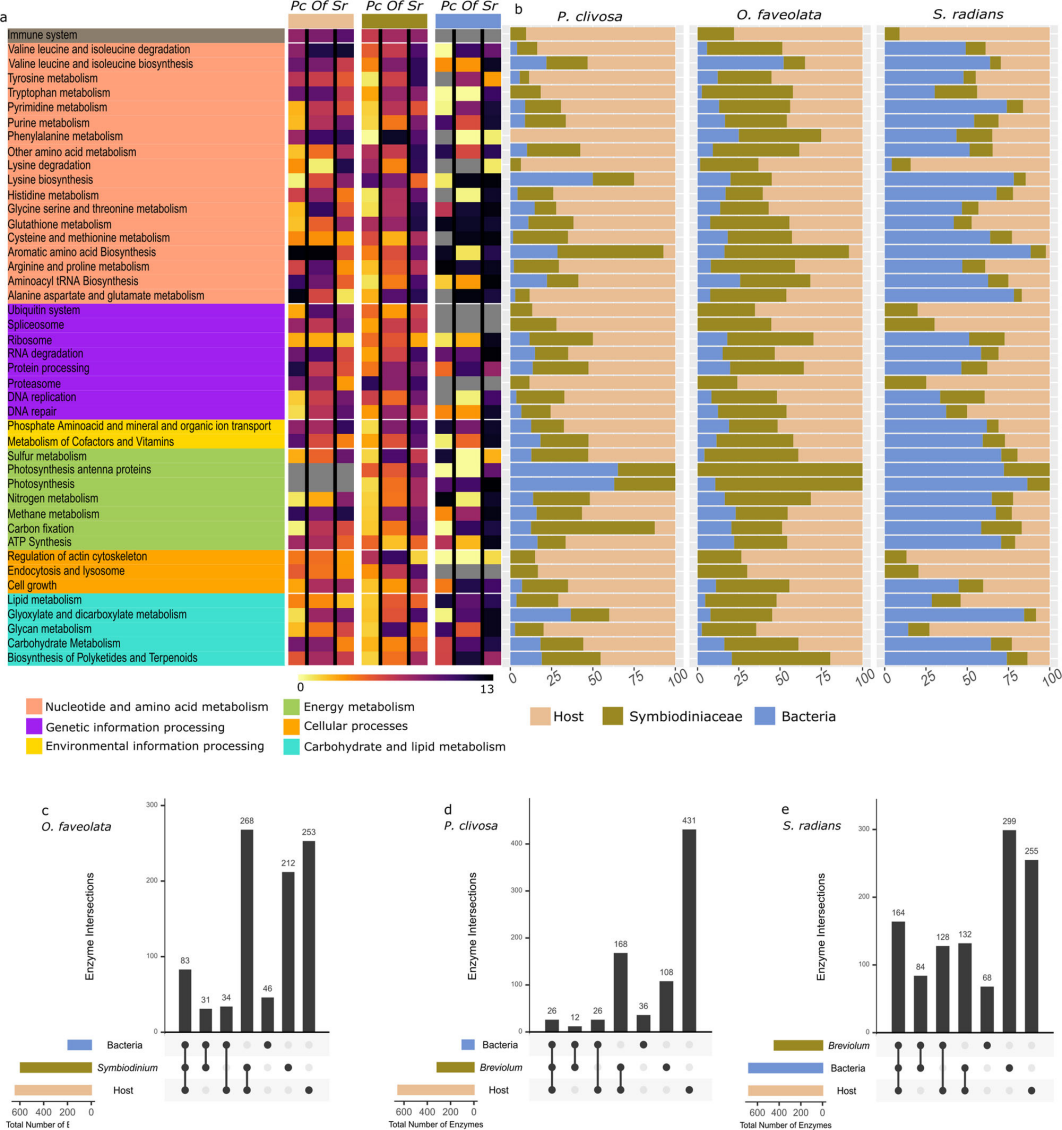
Quantitative Metabolic Fingerprint (QMF) and metabolic capabilities of holobiont members differ among coral species. The impact of heat stress from different holobiont members was assessed using QMF. **a** The fold change from ambient *vs*. heat-treated summed proportion of mapped reads falling into each of the KEGG pathways per species (*Pc* *P. clivosa, Of* *O. faveolata, Sr* *S. radians*) and normalized by the total expression of all KEGG pathways per holobiont member is depicted as a heatmap. **b** The metabolic capabilities are represented in a bar plot and were determined by calculating the proportion of transcripts derived from the sequenced metatranscriptomes falling into each KEGG pathway in a specific holobiont member and across coral species. (**c**, **d**) Comparative analyses of the shared and unique metabolic capabilities (annotated KEGG enzymatic reactions inferred in the metatranscriptome) within the holobiont members across coral species: **c** *O. faveolata*; **d** *P. clivosa*; **e** *S. radians*.

There is some evidence for coral-microbiome phylosymbiosis with specific bacterial members^49,50^. Since every coral holobiont has a characteristic physiological, transcriptional and evolutionary response to heat stress we sought to integrate the microbial communities’ metabolic response to elevated temperature. A vast majority of *P. clivosa* metabolic capabilities are dictated by the host, followed by its photosymbiont (Fig. 6B, C). *O. faveolata*, which is the species with the largest thermal susceptibility according to its photosymbiont loss under stress (Fig. 1E), derives a great percentage of metabolic capabilities from its photosymbiont *Symbiodinium* A3 in combination with the host capacities. In *S. radians* a higher proportion of metabolic capabilities are derived from its microbial communities (Fig. 6B, C). As a proxy for the metabolically expressed capacities, we calculate the Quantitative Metabolic Fingerprint (QMF)^51^ for *O. faveolata, S. radians,* and *P. clivosa* holobiont members (i.e., host, photosymbiont, microbiome) (Fig. 6A). We observed a divergent baseline of expression across members of the three holobionts, with regards to QMF. Thus, each holobiont starts with different metabolic capabilities under ambient conditions creating diverging trajectories during thermal challenge (Fig. 6A).

*S. radians* holobiont members had the greatest QMFs capacity. In agreement with the highest proportion of deduced bacterial metabolic capabilities, there was a corresponding increment in overall expressed transcripts with metabolic function (Fig. 6A). The QMF from the associated *Breviolum* B5 was higher for most of the metabolic modules when compared with the other Symbiodiniaceae (i.e., sustaining an active metabolism). This suggests a holobiont coordinated response where all the different members are actively engaged towards a symbiosis paired with subtle attenuated Symbiodiniaceae expression (Fig. 6A–C). *Symbiodinium* A3 QMF follows a trend similar to that of the host during heat stress, however certain metabolic modules like purine, glutathione, and carbohydrate metabolism, do not follow the trajectory of the host as seen in other species. This is likely due to adjustments being made by the host and the algae to overcome the disruption of symbiosis, which is congruent with the observed algal cell loss. *P. clivosa* metabolic capabilities are contributed in higher proportion by the host, but paradoxically during heat stress the QMF is lower in many metabolic modules, as well as in the *B. faviinorum* counterpart, suggesting a less robust holobiont system highly sensitive under elevated temperature (Fig. 6A, B).

### Diversity in metabolic capabilities and redundancy contributes to holobiont robustness

We determined the shared of metabolic reactions among different members, host with Symbiodiniaceae, host and Bacteria, Symbiodiniaceae and Bacteria and multipartite (host, Symbiodiniaceae and Bacteria) as well as unique reactions performed in the holobiont (Fig. 6C). We found that the majority of metabolic capabilities in the *P. clivosa* holobiont are encoded by the host. The coral-algal set shared a good proportion of metabolic capabilities, their metabolic compatibility during heat stress is generally decoupled (Fig. 6A). The number of overlapping shared metabolic capabilities of *O. faveolata* with the algal symbiont is high, therefore, the host can compensate for some of the metabolic pathways rendered inactive by the photosymbiont. Although this holobiont had the most dramatic decrease in cell density (Fig. 1E), the QMF demonstrates that the remaining population of algal cells are still metabolically active. Furthermore, despite the large reduction in photosymbiont number and the consequent fast rise in the effective light absorption per photosymbiont (a*_sym_), *Symbiodinium* A3 in *O. faveolata* showed a robust response to light stress, as reflected by the relative smaller declines in *Fv/Fm* observed. In comparison, *Breviolum faviinorum* showed larger *Fv/Fm* reductions associated with much smaller increases in a*_sym_ originated from the cell loss experienced by *P. clivosa* during heat stress. *O. faveolata* can rely on establishing symbiosis with other Symbiodiniaceae during prolonged stress raising new trade-off scenarios as previously observed^15^. *S. radians* shows a contrasting trend, most of the reactions are redundant across the different members, an advantageous trait during stress. Bacterial diversity in the thermotolerant species *S. radians* is significantly higher ab initio when compared to the susceptible *O. faveolata* and *P. clivosa* (Supplementary Fig. 4). The bacterial diversity, bacterial metabolic capabilities, and transcriptional activity in the thermotolerant species, suggests a crucial role of coral microbiomes in holobiont metabolism which can lead to increased survival during stressful conditions.

To the best of our knowledge, we have uncovered more genes associated with a thermal stress response in coral holobionts than any previous study and we show the gene expression level adaptation for each one of those genes. Our differential expression analyses of both orthologues, and lineage-specific transcripts, revealed a non-conserved response. The documented divergent transcriptional endpoint across holobionts during a thermal challenge highlights the fact that each photosymbiont establishes a specific extended phenotype with the host. By focusing on the expression level variance using a phylogenetic framework, we captured evolutionary differences in gene expression along different branches of the Scleractinia and Symbiodiniaceae trees that are undetectable with conventional transcriptome analyses. We identified 30 conserved gene expression profiles across two members of the suborder Faviina in the Robust clade, a scleractinian branch that dates back at least 93 million years. We also identified lineage-specific adaptations for each coral and associated Symbiodiniaceae species. The conservation of these gene adaptations can now be tested within each of those genera (e.g., within the *Orbicella* species complex, or Symbiodiniaceae genera) or at higher taxonomic ranks (e.g., family) with confirmed monophyly. Our findings illustrate how knowing the evolutionary history of gene expression adaptation can help us better predict future outcomes under the unidirectional trends of climate change-induced natural selection. We have implemented novel variations (EVE-R and EVEReSt) of the Phylogenetic ANOVA to include the response to any given stimulus, making it a tool that can be universally used across taxa and under any treatment condition.

Many of these genes divergently transcribed in different coral lineages are directly involved in coral-algal symbiosis. Few of them have been previously identified as genetic markers correlated with stress tolerance in coral populations^52,53^. The divergence observed in transcriptional activity for those genes may provide a rapid mechanism for adaptation to environmental factors at the regulatory level^54^. Corals have experienced episodes of elevated temperatures through evolutionary time^55^, and those lineage-specific expression shifts could be key in setting the host-photosymbiont thermal thresholds. Whether variation in transcriptional regulation contributes to faster adaptation is a fundamental question to understand the potential of coral-algal evolvability under the face of global warming. Future population level studies need to consider the regulatory potential of cnidarian and photosymbiont genomes, to build a clear understanding of the adaptive potential of each species.

Thermotolerance can be conferred through multiple layers of adaptation (i.e., at the genomic and regulatory levels). The role and the possible contribution of each regulatory level to the acquisition of thermotolerance in corals is still poorly understood, as is the possible contribution of the microbiome^56,57^. Metabolic redundancy is key to holobiont robustness hinting at potentially different holobiont metabolic strategies (i.e., synergistic, competitive, one-way interactions) taking place during heat stress. Our data suggest that coral holobiont thermotolerance can be boosted by the redundancy of key metabolic pathways from different members though this is yet to be experimentally tested. We show that thermotolerant species harbor a stable and diverse microbiome translating as a vast array of metabolic capabilities remaining active during the thermal challenge, contrary to the susceptible species, where the bacterial activity and diversity are reduced ab initio. Our study corroborates the observations that specific coral-microbiome dynamics can lead to different outcomes when divergent holobionts are faced with environmental changes^58^. Coral robustness to heat stress depends on coral ability to minimize light stress to the symbiotic algae, and this can be achieved by different strategies, one of them is to prevent or minimize photosymbiont loss under stress.

Our results set the stage for a mechanistic understanding of how different holobiont members bestow robustness on the coral as a unit. This study highlights the power of comparative metatranscriptomics and multispecies comparisons to unravel the complex coral holobiont metabolic interactions, fundamental to better predict the species survival of these ancient holobiont interactions under the current global climate change threats.

## Methods

### Experimental approach

Samples of *O. faveolata, P. clivosa*, and *S. radians* were collected in the reef lagoon of Puerto Morelos, Mexican Caribbean, Quintana Roo, Mexico, in November 2008 (20°55'39.54'' N - 86°49'58.90'' W; Permit Registration MX-HR-010-MEX folio 016). Samples were transported to the mesocosm facilities at the Unidad Académica de Sistemas Arrecifales, Universidad Nacional Autónoma de México (UASA-UNAM), and placed in outdoor tanks equipped with running seawater supplied from the reef lagoon. Tanks were shaded with neutral mesh screens, allowing for the reduction of light intensity to levels similar to the collection depth (~ 37% of surface irradiance, Es). For all species, samples consisted of two fragments from 5 different colonies, which were distributed over two outdoor 152 L tanks (*n* = 6 specimens per species/per tank). The average flow rate in the experimental tanks was 1.45 L s^–1^, with a turnover rate of ∼ 105 min. Water temperature was maintained for 16 days at 28 °C ( ± 0.4 °C in November) after sampling using commercial aquaria heaters (Process Technology, USA), located in header tanks and connected to thermocouple sensors (J type, TEI Ingeniería, Mexico). Irradiance (μmol quanta m^–2^ s^–1^) and water temperature (°C) were continuously monitored using a cosine-corrected light sensor (LI-192) connected to a Data Logger (LI-COR 1400, Lincoln, NE, USA), and Hobo data loggers (Onset Computer Corporation, MA, USA). One of which was placed in a control tank and the other in an experimental tank at Instituto de Ciencias del Mar y Limnología, Universidad Nacional Autónoma de México. Each species was represented by three biological replicates in the control tank paired with three replicates in the experimental tank.

Following the 16 days of acclimation, heaters were turned on for one of the tanks. The temperature in the control tank remained at ~ 28 °C, while in the heat-treatment tank was increased to 32 °C ± 0.34 at day 0. Photosynthetically Active Radiation (PAR) measured at noon was average 230 ± 57 μmol quanta m^–2^ s^–1^ for both tanks. As no signs of changes in coral color were observed for 7 days, the water temperature was increased to 34 °C ± 0.30 and maintained for 2 days until the end of the experiment. The stress period lasted a total of 9 days. During the stress treatment algal physiology was monitored daily using diving pulse amplitude modulated (PAM) fluorometry (Heinz-Walz GmbH, Germany). Measurements of the maximum photochemical efficiency of photosystem II of the photosymbiont (*Fv/Fm*) were performed at dusk, as this time allows for detection of the maximum value of diurnal variation in *Fv/Fm*^59^. At the end of the experiment, fragments were immediately flash frozen and preserved in liquid nitrogen, and kept at – 80 °C until further processing. Symbiodiniaceae cell density within the control and experimental coral fragments was measured for a defined area of coral tissue. For the same area and previously to freeze the samples, the optical properties of each organism were characterized according to Scheufen et al.^60^ and Vásquez-Elizondo et al.^61^, using reflectance determinations. Reflectance at the peak of chlorophyll-a absorption at 675 nm (R_675_) was selected to estimate the variation of the effective light absorption by Symbiodinaceae *in hospite* (a*_sym_), according to the following equation: *a*∗_sym_ = [De_675_/photosymbiont density] · ln(10) (see refs. ^17,60,62^). De_675_ refers to the estimated absorbance*^62,63^ and was calculated as [De_675_ = log(1/R_675_)]. This parameter, a*_sym_, can be used as a proxy for the variability among species and coral phenotypes in the efficiency for solar energy collection of Symbiodinaceae *in hospite*^62^. The parameterization is similar to the calculation of the specific absorption coefficient of chlorophyll-a developed for phytoplankton^64–67^ and previously determined for symbiotic tissues by^68–70^, and more recently for the whole coral structure by^12,60,62^. The quantification of a*_sym_ allows a description of the magnitude of the effective area expanded by each photosymbiont cell *in hospite* for solar energy collection and has the units m^2^ sym^–1^. Differences among control and heat treatments were analyzed using a *t*-test for all the tested parameters. One-way ANOVA and Post-hoc Tukey HSD tests were used to detect significant differences on all the photophysiology traits studied among Symbiodiniaceae species in the response to thermal stress. An ANCOVA analysis allowed for the assessing the differences between species in the linear relation of log-log transformed a*_sym_ and symbiont density. All statistical analyses were conducted using R (v. 3.6.0) and using the car library to implement type III errors.

### RNA library preparation

Coral tissue from three biological replicates for the control and treatment were ground to a fine powder in liquid nitrogen, after skeleton removal on dry ice using a chisel and hammer. We extracted total RNA using the mirVana miRNA Isolation Kit (Life Technologies) according to the manufacturer’s protocol, with the following modification. In order to disrupt the photosymbiont cell wall, for all the collected and grinded corals, we added 0.2 grams of coral fine powder into a tube containing of 0.1 mm silica beads and we added immediately the Lysis/Binding Buffer. We used a bead better for 30 s and we let sit the sample for 1 min on ice, this procedure was repeated three times in order to achieve cell wall and cell lysis in the sample. The resultant supernatant was used for the subsequent steps using the manufacturer’s protocol. We purified and concentrated the RNA by using the RNA Clean and Concentrator kit (Zymo Research, Irvine, USA). RNA yield was assessed on a Nanodrop and Qubit using 2.0 RNA Broad Range Assay Kit (Invitrogen). We verified RNA quality using the Agilent Bioanalyzer 2100 (Agilent Technologies, Santa Clara, USA). The RNA samples had an RNA Integrity Number (RIN) between 8.3 and 9.5 (Supplementary Table 1). Total RNA samples (100 ng per sample) were sent for metatranscriptome sequencing to the JGI, in Walnut Creek, California. RNA integrity and quality were again reassessed prior to the subsequent ribosomal RNA depletion and mRNA enrichment from whole holobionts employing the Ribo-Zero kits (Epicenter). Following the RiboZero protocol, mRNA was converted to cDNA and amplified. The libraries were sequenced on the Illumina HiSeq 2000 platform using 2 × 151 bp overlapping paired end reads.

### Metatranscriptome assembly, quantification, and expression analyses

The raw reads were quality-trimmed to Q10 adapter-trimmed and then filtered for process artifacts using BBDuk^71^. Ribosomal RNA reads were removed by mapping against a trimmed version of the Silva database using BBmap (http://sourceforge.net/projects/bbmap)^71^. A summary of metatranscriptome libraries sequencing statistics is provided in Supplementary Table 5. In order to generate a de novo reference metatranscriptome for each holobiont species, the cleaned and filtered reads from the replicates from control and treatment fragments per each species were pooled and assembled using Trinity (v2.4.0)^72^, generating three metatranscriptomes (*O. faveolata, S. radians*, and *P. clivosa)*.

In order to identify and separate the different members (i.e., host, photosymbiont and bacterial associated communities) of the holobiont per each metatranscriptome species assembly, a blastn search using the megablast-dc algorithm^73^ was performed. A local database built with available cnidarian genomes (*Nematostella vectensis*^74^*, Acropora digitifera*^75^*, Orbicella faveolata*^3^, and *Exaiptasia pallida*^76^), Symbiodiniaceae genomes (*Breviolum minutum*^77^*, Fugacium kawagutii*^78^, and *Symbiodinium microadriaticum*^79^) as well as *Breviolum* transcriptomes^80^ was used for the aforementioned blastn^73^. To identify bacterial species represented in each coral holobiont metatranscriptome we computed a blastx alignment with DIAMOND (v0.8.22)^81^ against nr. The results were inspected, and bacterial taxa were filtered using MEGAN (v5)^82^. The bacterial taxa identification was used to retrieve full bacterial genomes corresponding to the blastx matches from NCBI. We added all these bacterial genomic sequences obtained at the previous step to the coral host and photosymbiont genome database and we performed the blastn search using the MEGABLAST-DC algorithm^61^ to select high confidence bacterial transcripts. Transcriptome completeness was assessed using the bioinformatics tool BUSCO (Benchmarking Universal Single-Copy Orthologues) (v4.1.4)^83^ using the Metazoa odb9 set against the three separated host transcriptomes for the three coral species, and by using the Eukarya database against the three associated- Symbiodiniaceae transcriptomes. We determined transcriptomes statistics such as N50 and mean length by using abyss-fac (v2.1.5)^84^. Summary statistics for each assembly are provided in Supplementary Note 7, Supplementary Fig. 1.

Kallisto (v0.43.0)^85^ was used to perform a pseudoalignment and to quantify transcript abundances. Kallisto quantification does not rely on an available genome reference, instead, it uses de novo assembled reference transcriptomes. A comparison of counts per million, a correlation matrix and principal component analysis among samples was done for quality check of the replicates per species. Expression analysis was performed using the DESeq2 software (v1.16.1)^86^. Differential Expressed Genes (DEGs) were defined by using both a strict cutoff threshold of FDR < 0.001 and a flexible FDR < 0.01, and – 2 < log Fold Change > 2. Amino acid sequences were predicted from the Coral host and Symbiodiniaceae transcriptomes by using Transdecoder (v2.0.1)^87^. Orthologous groups of protein sequences amongst the three coral and their associated three photosymbiont species were determined with the OrthoFinder (v2.4.0)^16^ bioinformatics tool, using default parameters. Using reciprocal best-hits via BLAST all-v-all algorithm, Orthofinder determined the number of conserved putative orthologues among the three coral and three photosymbiont species. By a custom python script, the ortholog differential gene expression (logFC) was retrieved for each expression dataset per species against the other two species.

### Functional Gene Ontology (GO) Summaries

Gene ontology (GO) annotations for genes were obtained through eggNOG-mapper (v2.0)^88,89^. The GO annotation was used to identify gene functional annotation. GO enrichment analysis was performed using RBGOA^90^, an R package available on GithHub (GO-MWU, https://github.com/z0on/GO_MWU) enabling us to measure significance on a continuous value by performing Mann–Whitney U tests. Signed log *p*-values from the previous differential expression were used as input for the enrichment analyses. We performed this analysis for the coral and photosymbiont taxa, enriched GOs per host or photosymbiont were filtered based on FDR < 0.05. Conserved enriched GO terms across host and photosymbiont species were determined using set operations.

### Symbiodiniaceae genotyping

Approximately 0.2 g of coral fine powder obtained at RNA library preparation previous step was used as the input material for the DNA extraction. DNA was extracted using the Mo Bio Laboratories PowerFecal DNA Isolation Kit, following the manufacturer’s standard procedure. 30 ng of total DNA was used to amplify the Symbiodiniaceae ITS2 region using the oligonucleotide primers described in Pochon et al.,^91^ (Supplementary Table 8). Each 30 µL PCR reaction was prepared with 10 µL GoTaq (Promega), 3 µL forward primer (10 µM), 3 µL reverse primer (10 µM), 2 µL template DNA, and 12 µL PCR-grade water. PCR amplifications consisted of a 5 min denaturation at 94 °C; 30 cycles of 40 s at 95 °C, 2 min at 59 °C and 1 min at 72 °C; and 5 min at 72 °C. These amplicons were sequenced using Illumina MiSeq 250 bp PE libraries, targeting ~ 50 K reads at the Genomic Sequencing and Analysis Facility, University of Texas. A quality check on the sequencing data was performed using FastQC (v0.11.2)^92^. We built a script to run a pipeline processing all the samples at once. Pair end data from each sample were merged using FLASH (v1.2.11)^93^ with overlapping of 300 nucleotides. A quality filtering on the extended amplicons was performed using USEARCH (v6.1)^94^ (fastq_filter option). For finding the unique OTUs, identical sequences were removed with the USEARCH function for dereplication (derep_fulllength). OTUs were clustered from the sequences in all samples with 97% similarity through the cluster_otus function in USEARCH. Then all the sequences for each colony sample were mapped to the OTUs to assess the specific abundance using usearch_global. Absolute abundances were normalized by calculating the OTU abundance per coral sample by the total abundance ratio. OTUS with < 20 reads in a coral species were discarded. OTU identification was done using blastn against a curated database with sequences from *Cladocopium* published in^95^, and other genera including, *Symbiodinium, Breviolum, Durusdinium*, and *Effrenium* from ref. ^96^.

### 16 S rRNA analyses

Approximately 0.2 grams of coral fine powder obtained at the RNA library preparation previous step was used as the input material for the DNA extraction. DNA was extracted using the Mo Bio Laboratories PowerFecal DNA Isolation Kit, following the manufacturer’s standard procedure. 10 ng of total DNA was used to amplify the rRNA DNA 16 S region using the oligonucleotide primers using the 515 F/806 R primer pair that targets bacterial and archaeal communities for V4-V5 (16 S) described in Parada et al.,^97^ (Supplementary Table 8). Each 25 µL PCR reaction was prepared with 10 µL 5Prime HotMaster Mix (VWR International), 0.5 µL forward primer (10 µM), 0.5 µL reverse primer (10 µM), 1 µL template DNA, and 13 µL PCR-grade water. PCR amplifications consisted of a 3 min denaturation at 94 °C; 30 cycles of 45 s at 94 °C, 60 s at 50 °C and 90 s at 72 °C; and 10 min at 72 °C. These amplicons were sequenced using Illumina MiSeq 250 bp PE libraries, targeting ~ 200 K total reads per sample (Supplementary Table 7). Sequencing was done at the JGI. We used QIIME2 (v2017.7)^98^ and DADA (v2)^99^ to denoise the amplicon sequences to determine the amplicon sequence variants (ASV) to process all 16 S rRNA libraries. We estimated the alpha Shannon diversity index, and Chao richness by using the phyloseq (v1.32.0)^100^, and microbiome R packages (v1.10.0) (https://microbiome.github.io/microbiome/). vegan R package (v2.5-6)^101^ was used to compute and visualize the rarefaction curves for each sample.

### Quantitative metabolic fingerprinting (QMF)

The QMF was calculated by normalizing by the median expression at the module level (KEGG metabolic pathway) to the total mapped reads in each metatranscriptome^51,102^. Briefly KEGG (Kyoto Encyclopedia of Genes and Genomes) pathways and KEGG Ortholog (KO) annotations were assigned to assembled transcripts for each metatranscriptome using MEGAN (v5)^82^. We only considered genes with at least 10 Transcripts Per Million (TPM) in at least 2 control and/or treatment samples. We calculated the TPM mapping for each transcript mapping to a certain KEGG module for the whole holobiont. The number of transcripts belonging to each KEGG module per holobiont member (Host, Symbiodinaceae, and Bacteria) was calculated.

### Metabolic capabilities and redundancy analyses

The transcriptomes of each member for the three coral species were annotated retrieving the Enzyme Commission (EC) Number from the KEGG database using MEGAN (v5)^82^. Using a customized Perl script EC total reactions were quantified. The metabolic reactions were classified as multipartite if all the holobiont members (Host, Symbiodiniaceae, and Bacteria) could perform the same metabolic reaction, bipartite if at least the reaction is present in two members (Host-Symbiodiniaceae, Host-Bacteria, Symbiodiniaceae-Bacteria) and unique if a single member could perform a specific reaction. Gene expression (Transcript Per Million) of these enzymes per taxa in the three coral holobionts was quantified and classified per category: multipartite if all the members are capable to perform an enzymatic reaction; dipartite if at least two members perform the same reaction or unique when just one member has the capability to perform a specific enzymatic reaction.

### Expression variance and evolution model

Through RNA-seq is possible to test the variation in gene expression among species and within species assuming gene expression as a quantitative trait. By considering a phylogenetic ANOVA and the Expression Variance and Evolution Model EVE^38^ that parametrizes the ratio (β) of population to evolutionary expression level, it is possible to detect genes with ratios between lineage expression divergence, as well as within species expression diversity. If there is stabilizing selection or no selection is acting in the expression (quantitative trait) then β will remain constant. If this ratio of within-species expression variance to across species expression evolutionary variance is higher and the variance is more within versus between lineages, there is diversity of expression. When β is lower for genes with an increased variance between lineages, lineage specific expression shifts can be tested, i.e., expression divergence. Here we use EVE to identify genes with high expression divergence in coral hosts and their dinoflagellate photosymbiont as candidates for expression level adaptation.

A gene expression matrix with the control samples for a core conserved gene among coral and Symbiodiniaceae species defined by orthology was generated by using Agalma (v0.5.0)^103^. The former phylogenomic algorithm was also used to build the coral and Symbiodiniaceae phylogenetic tree needed to run the EVE model. The EVE model calculates a likelihood ratio test (LRT) test to examine the null hypothesis of a β ratio for a given gene equal to β for all the tested genes. χ2 distribution is expected for the LRT statistic under the null model. By using a cutoff value of p < 0.05, candidate genes were determined for lineage-specific shifts in expression vs. expression diversity gene candidates deviated from the null distribution. Our first approach, EVE-R (EVE-Response) considered candidate genes determined by the model as divergent expression with a different expression baseline between species from control samples. Genes were filtered by selecting those for which expression changed after the heat treatment. Our second approach EVEReSt (EVE in response to stimulus) took into account the intrinsic response to heat stress by subtracting the expression values from the treatment to the control samples per species. After running the EVE model, the divergent expression gene candidates were considered as likely thermal adaptive genes.

Gene ontology enrichment analysis was performed using RBGOA^90^. Since we wanted to test our specific EVE ortholog gene expression as the overall set of genes and determine enriched GOs for the adaptive gene expression candidates, a Fisher exact test was performed. Adaptive gene expression candidates were labeled as 1, and the rest of the orthologues expression was set to 0. This binary list was used as input for the enrichment analyses. This analysis was performed for the coral host and photosymbiont taxa.

### Reporting Summary

Further information on research design is available in the Nature Research Reporting Summary linked to this article.

## Supplementary information

Supplementary Information

Description of Additional Supplementary Files

Supplementary Data 1

Supplementary Data 2

Supplementary Data 3

Reporting Summary

## Data Availability

All raw sequencing data that support the findings of this study have been deposited in the National Center for Biotechnology Information Sequencing Reading Archive (SRA) and are accessible through the SRA Series accession numbers: “SRP123687”, “SRP123688”, “SRP123689”, “SRP123701”, “SRP123703”, “SRP123705”, “SRP123711”, “SRP123713”, “SRP123717”, “SRP123716”, “SRP123715”, “SRP123719”, “SRP123718”, “SRP123722”, “SRP123725”, “SRP123724”, “SRP123726”, “SRP123727”, “SRP325190”, “SRP325186”, “SRP325185”, “SRP325188”, “SRP325181”, “SRP325183”, “SRP325199”, “SRP325197”, “SRP325194”, “SRP325196”, “SRP325195”, “SRP325192”, “SRP325184”, “SRP325198”, “SRP325193”, “SRP325201”, “SRP325203”, “SRP325182”. Additionally all raw and filtered metatranscriptomic and 16 S rRNA amplicon sequencing data, statistics, and quality sequencing reports for *Orbicella faveolata*, *Siderastrea radians* and *Pseudodiploria clivosa* are available at the US Department of Energy’s Joint Genome Institute (JGI) Genome Portal (https://genome.jgi.doe.gov/portal) with accession numbers: 1086628, 1086630, 1086632, 1086634, 1086636, 1086638, 1086592, 1086594, 1086596, 1086598, 1086600, 1086602, 1086622, 1086624, 1086626, 1086616, 1086618, 1086620, 1101076. (Community Sequencing Project 1622: How do coral hosts communicate with their associated microbial community?). Data generated in this study have also been deposited in the Dryad repository (10.5061/dryad.k3j9kd57b). Source data are provided with this paper.

## Source data

Source Data

## Code availability

The code to replicate our analyses is available in the Zenodo repository (10.5281/zenodo.5310663).
