# Supplementary material for: Elucidating gene expression adaptation of phylogenetically divergent coral holobionts under heat stress
Source: Nat Commun. 2021 Sep 30;12:5731. doi: 10.1038/s41467-021-25950-4 (PMC8484447; doi:10.1038/s41467-021-25950-4)
Supplement: Supplementary file 1 — Supplementary Information [file 41467_2021_25950_MOESM1_ESM.pdf]

Supplementary Information

Elucidating gene expression adaptation of phylogenetically divergent coral holobionts under heat stress.

Avila-Magaña V, *et al.*, 2021

Supplementary Notes

1. Symbiodiniaceae physiology reveals a distinctive response to heat stress

After 9 days of heat exposure *O. faveolata-Symbiodinium* A3 and *P. clivosa-B. faviinorum* showed a drastic reduction in symbiont density (t-test; p=0.0005866; p=0.01263 respectively), in comparison with control organisms maintained at 28ºC. In contrast, *S. radians-Breviolum* B5 exhibited a non-significant reduction in symbiont density after the heat stress exposure (Fig. 1D, Supplementary Table 3,4).

A decrease of PSII maximum quantum yield efficiency (*Fv/Fm*) during heat stress (34ºC) indicates significant increases in light stress for Symbiodiniaceae *in hospite* during the elevated temperature treatment, which may or may not result in photosynthesis impairment[1,2], but certainly increases the costs of repair and maintenance of photosynthetic activity. *Fv/Fm* revealed a statistically significant decrease in *Symbiodinium* A3 after 2 days of heat stress, whereas *Fv/Fm* in *B. faviinorum* and *Breviolum* B5 decreased significantly after 3 days (Fig 1C). After 9 days, a significant decline in *Fv/Fm* on the heat-treated samples showed major differences between coral species (ANOVA, p=4.59e-06, Supplementary Table 9, Fig. 1C). By the end of the experiment, maximum quantum yield in *B. faviinorum* dropped abruptly, in *Symbiodinium* A3 it decreased to a lesser degree, and in *Breviolum* B5 it only diminished slightly (Fig. 1C).

The large values of a^*_{sym} determined for *Symbiodinium* A3 in *O. faveolata*, respond to the low number of Symbiodiniaceae cells that this species had at the time this experiment was performed (November 2008). This low symbiont content does not correspond with the large pigmentation and symbiont species that *O. faveolata* tends to associate with in this area of the Caribbean[2] and elsewhere[3]. The reef lagoon of Puerto Morelos experienced two consecutive events of elevated temperatures during the previous summer, and *O. faveolata* was found particularly paled during the summer of 2007[4]. Therefore, the process of recovery after both bleaching events could explain the low number of photosymbionts that *O. faveolata* harbored in our experimental study in comparison with other analysis performed in the same area[2].

Based on ITS2 rRNA Symbiodiniaceae sequencing, we found that after 9 days the photosymbiont composition did not change between control and heat-treated colonies, *P. clivosa* hosted *Breviolum faviinorum* and *Breviolum* B5 were associated with *S. radians* whereas *O. faveolata* associated with *Symbiodinium* A3.

2. Heat stress induces specific gene expression profiles in corals

The coral host species with the highest number of DEGs was *P. clivosa*, followed by *S. radians* and *O. faveolata* (633/568/366 respectively; FDR =0.001 (Supplementary Data 1). The DEGs in *P. clivosa* had an average fold change of 3.66 (range: 2.00–11.42) for up-regulated and −3.62 (range: −2.01 to −9.97) for down-regulated transcripts. Similarly, the *O. faveolata*'s fold change average was similar to the *P. clivosa* upregulated group at 3.29 (range: 2.00-11.29) and for down-regulated genes the average was –3.76 (range: -2.00 to -25.11). Strikingly, the *S. radians* average fold change for up-regulated genes was 6.59 (range: 2.23-13.02) and -6.60 (range: -2.01 to -21.55).

A set of genes are upregulated in *P. clivosa* when compared to the ortholog profile expression from the other species. For example, during the temperature challenge, the over expression of the *TNFR* 8 and 10 is less prominent in *O. faveolata* and higher in *P. clivosa* (Supplementary Data 1). When considering the Faviina expression profiles as reference to assess the ortholog

expression in *S. radians*, we found that a vast majority of genes is not even differentially expressed, followed by a set of DEGs that aren't statistically significant and in some cases with an opposite transcriptional profile (Supplementary Fig.2 A-B). A gene encoding for an allene oxide synthase-lipoxygenase is significantly overexpressed in *P. clivosa* when compared to *S. radians*, where it is under expressed (Supplementary Fig.2C). This protein plays a role in jasmonic acid biosynthesis in plants and has been studied in octocorals due to its catalase activity[5]. *S. radians* significant DEG profiles in general are not conserved with the Faviina orthologues (Supplementary Fig.2C).

The KEGG phagosome category was an enriched in all the species but was under-expressed for *S. radians* and *O. faveolata*, and over-expressed for *P. clivosa*. Within this category the shared macrophage scavenger receptor 1 (K06558) and the macrophage receptor with collagenous structure (K13884) genes followed opposite patterns of expression between *P. clivosa* (logFC= 2.6 (K06558); 2.2 (K13884)) and *O. faveolata* (logFC= -3.6 (K06558); -6 (K13884)).

As not all the DEGs in each species were part of the KEGG enrichment analyses, we identified a subset of those DEGs previously described in coral transcriptomic studies of heat stress and bleaching. These DEGs involved in heat shock response, oxidative stress, immunity, ion transport, cytoskeleton and extracellular matrix, cell adhesion, biomineralization, protein folding and processing, apoptosis, and with miscellaneous activity[6] were found in all three coral hosts. The expression profiles of these DEGs are not consistent with a conserved heat stress response across scleractinian corals. In addition to previously described bleaching gene markers (reviewed in Louis et al., 2017[66]; Maor-Landaw and Levy, 2016[6]), we identified to the best of our knowledge a novel subset of DEGs present in all coral hosts that to our knowledge has not been implicated before as a part of heat stress or bleaching (Supplementary Fig.3D). These shared DEGs did not elicit the same transcriptional profiles across taxa. For example, *NFX1 type zinc finger contains protein 1* which is overexpressed in *O. faveolata* and displays an opposite trend in *S. radians*. This protein has been recently implicated in *C. elegans* as a factor required for epigenetic inheritance to guarantee an equilibrated amplification of small RNA signals and it has been hypothesized that it may establish epigenetic programs that allow a rapid adaptation to environmental perturbations in the wild[7]. Further studies on different coral populations and species to understand the molecular mechanisms of epigenetic inheritance and their role of this protein during bleaching are warranted.

3. Symbiodiniaceae expression profiles reveal differential regulation in key metabolic and cellular pathways

The DEGs in *B. faviinorum* had an average fold change of 4.83 (range: 2.083 – 9.689) for up-regulated and −3.41 (range: −2.00 to −8.56) for down-regulated transcripts. Similarly, the *Symbiodinium A3* DEG fold change for the upregulated group was 4.61 (range: 2.06 – 9.95) and for down-regulated genes the average was -4.87 (range: -2.00 to -10.47). The *Breviolum B5* DEG average fold change was slightly lower for both up- (3.30, range: 2.13 - 8.45) and down-regulated genes (-3.64, range: -2.01 to -10.19).

The transcriptional profile of each Symbiodiniaceae species is unique. For instance, in *B. faviinorum* a nitrate transporter (*NRT2*) is differentially under expressed during heat stress. Sproles et al., 2015[11] identified this transporter homolog sequences in *Symbiodinium*. Our orthology analyses also found this transporter in *Breviolum* B5 and in the *Symbiodinium* A3 (Fig. 3B-C and Supplementary Data 1). Symbiodinaceae *in hospite* relies heavily on host ammonium uptake and translocated as the main nitrogen source instead of nitrate[12]. The

transport of nitrate via NRT2 seems unlikely since a structural analysis predicted its ligand as monoacylglycerol, and since the 3D structure resembles a bacterial glycerol-3-phosphate transporter[11]. Glycerol amounts and its release during osmotic stress in *Symbiodinium* in culture and during heat stress *in hospite* (*A. aspera* 6 and 8 d) have been documented and suggested as a metabolite conferring thermotolerance[13,14]. If monoacylglycerol (MAG) is transported by NRT2, during elevated temperature this transporter is under-expressed to increase MAG accumulation. Evidence from our evolutionary analyses indicates a divergent expression between lineages of the monoacylglycerol lipase (*MAGL*), an enzyme that cleaves this molecule in order to produce glycerol. Increased expression of *MAGL* under heat stress in the two *Breviolum* species when compared with the *Symbiodinium* A3 (Fig. 5) indicates an increase of glycerol production which may be also involved in lipid signaling during increasing temperature.

Symbiodinium A3 exhibits a general stress response of downregulation of metabolic and cellular processes, such as ion transporters (*zupT*), exocytosis/vesicular transport (*VPS35*) (Fig. 3B). VPS (vacuolar protein sorting proteins) and syntaxins have been hypothesized to be involved in symbiont shuffling and symbiont exit from the host during coral bleaching[15]. Interestingly, a slightly different transcript coding for *VPS35* displays an opposite trend being over expressed (logFC =8.4), in a similar fashion as *VPS53A*, highlighting the complexity of the transcriptional regulation of the SNARE processes during host-symbiont cellular interactions. Signal transduction regulates key cellular and metabolic pathways during heat stress. This cascade, however, is not well characterized in Symbiodiniaceae. We found a GABA receptor overexpressed solely in *Symbiodinium* A3-*O. faveolata* (absent in the other Symbiodiniaceae transcriptomes) (logFC= 2.44). This receptor is a component of a heterodimeric G-protein coupled receptor for GABA. This metabolite has been involved in pH and osmoregulation, used as an antioxidant and cell signaling[16]. Recently the receptor and GABA have emerged as part of a larger module in the response to biotic and abiotic stress in plants and diatoms[17]. We hypothesize that this receptor may be key to regulate and orchestrate Symbiodiniaceae stress response.

Another gene overexpressed and present uniquely in *Symbiodinium* A3 is a glutamyl tRNA (Gln) amidotransferase (*GATA*, logFC= 2.12). During heat stress translation machinery is prone to errors, and misacylated tRNAs may occur[18]. *GATA* upregulation likely ensures the proper tRNA loading ultimately to maintain the protein synthesis.

In the profile expression of thermotolerant symbionts, *Breviolum* B5 mounted a reduced specific response that seems effective to contend with the heat stress. In general, this strategy consists in the downregulation of genes involved in ion and amino acid transport. Decreased expression of ZIP transporters (Zinc (Zn^{2+})-Iron (Fe^{2+}) Permease (ZIP) Family) in this symbiont hosted by *S. radians* as well as in *O. faveolata* symbionts highlight the importance of metal homeostasis during heat stress. DeSalvo, et al.[19] reported a homolog zinc transporter gene downregulated in the *Acropora palmata* associated *Symbiodinium* during bleaching induced by darkness. Zinc is an essential trace metal since it is involved in many biological processes such as redox biochemistry, DNA stability, gene expression, autophagy and apoptosis[20-22]. Carbonic anhydrase has a zinc cofactor that catalyzes the reversible conversion of HCO_3 and CO_2, this CO_2 is then concentrated at the active site of Rubisco in order to be fixed during the Calvin cycle. Furthermore, zinc accumulation and uptake are greater in the host and *Symbiodinium* in symbiosis than aposymbiotic anemones[23] concordantly with the differential expression of different zinc transporters upregulated in symbiotic anemones[24]. An impairment between host and symbiont zinc absorption may occur during heat stress and

bleaching, photosymbionts may accumulate metals and nutrients to sustain the energetic budget imposed by the symbiosis.

Over expressed *flavodoxin* has been documented in diatoms during iron limitation, to overcome the iron expense imposed by the synthesis of chloroplast ferredoxin[25]. We reported a unique flavodoxin gene present and downregulated in *Breviolum* B5, which indicates that during elevated temperature the thermotolerant symbiont is not iron limited. We corroborate this notion by observing the constitutively expression of ferredoxin genes irrespectively of the temperature increase. Furthermore, *in silico* prediction of this flavodoxin cellular location indicates likely to reside in the cytoplasm, suggesting a specific role other than electron transference during photosynthesis.

B-hexosaminidase is upregulated as well in *Breviolum* B5. The specific role of this enzyme during heat stress or symbiosis is unknown, but it has been shown to have diverse functions. In the protist *Entamoeaba hystolitica,* it acts as a pathogenicity factor by cleaving the terminal N-acetyl-galactosamine (GalNAc) residues from the intestinal epithelial surface after lectin-mediated binding[26]. In mycorrhizal symbioses, the GalNAc residues residing in the cell wall have been described as a symbiont associated molecular patterns recognized by the plant. These residues may function as a mediator of the host cell-photosymbiont cell recognition and attachment. Lundgren, et al.[27] identified this gene as a pre-selection candidate where Single Nucleotide Polymorphisms (SNPs) correlated against a temperature gradient on different *Pocillopora damicornis* populations across the Great Barrier Reef, suggesting that this gene likely plays a role in heat stress tolerance within coral populations.

During heat stress, alternative metabolic pathways may be activated to sustain the symbiosis nutritional demand. 6-phosphogluconolactonase, an enzyme of the pentose phosphate pathway is over expressed in the thermotolerant *Breviolum* B5. There is some evidence that this enzyme is involved in commensal and pathogenic *Enterococcus faecalis* interactions with the tobacco hornworm *Manduca sexta*[28]. The pentose phosphate pathway is a source for the synthesis of aromatic amino acids and nucleotides. This pathway is key for the robust response mounted by *Breviolum* B5 since it is a metabolic redox sensor that generates the reducing equivalent NADPH, preventing oxidative stress by maintaining the pool of reduced glutathione (GSH), via glutathione reductase (GR). A previous study with Symbiodinaceae species in culture showed that a thermosensitive *Breviolum* B1 on a sub-lethal temperature increases the activity of GR, contributing to the availability of free glutathione notwithstanding even with increased ROS[29]. While this stress tolerance mechanism malfunctions under excessive heat in *Breviolum* B1 species, a thermotolerant *Fugacium* F1 species had an increase on GR activity irrespectively of the heat treatment, highlighting the importance of a redox balancing mechanism conferring thermotolerance[29]. Taken together, these results highlight the unique trajectories followed by each photosymbiont species and the key strategies to sustain symbiosis under adverse temperature increases.

4. Baseline expression adaptation among coral and symbiont species changing during heat stress (extended)

We identified orthologues common across all three host and symbiont species (1,579 and 1,419 respectively). There are candidates for expression level adaptation in the *O. faveolata- P. clivosa* host lineage (Fig. 4), such as *COPD* and *COPE* (coatomer subunit delta and epsilon) including genes putatively related to cycling between the endoplasmic reticulum (ER) and Golgi apparatus during heat stress, increasing the influx of damaged or misfolded proteins into the ER. Similar results have been found in the symbiotic anemone *Exaiptasia pallida*, where

coatomer protein was greatly elevated in heat-shocked conditions (33.5ºC~24h)[30]. This gene was already expressed in control *O. faveolata* and *P. clivosa* samples, which may reflect that these species live at a very fragile threshold of stress. In *S. radians*, COP (delta/epsilon) is not expressed either before or after stress, possibly indicating life at a closer to optimum temperature.

The transcriptional trajectory of a catecholamine receptor for octopamine (*OCTR*) is conserved in the Faviina lineage. The susceptible and intermediate species *P. clivosa* and *O. faveolata* expressed the octopamine receptor, which is not expressed in *S. radians* during heat stress. Under this environmental challenge, corals may experience starvation due to photosymbiont cell loss[31]. In other invertebrates such as worms, flies and snails; octopamine, an analogue of norepinephrine, is related to starvation[32,33]. Upon starvation, an increase in octopamine also triggers lipolysis to balance energy homeostasis[32]. Upon octopamine receptor stimulation, cAMP Response Element-Binding Protein (CREB) is activated. Our analysis detected *CREB3* as a candidate for adaptive response. This gene is upregulated in *P. clivosa* and *O. faveolata*. During ER stress response, CREB3 activates transcription of unfolded protein response (UPR) target genes[34] and it may also be involved in regulating vesicle trafficking from the ER to the Golgi[35,36]. We hypothesize that CREB3 may be activated via OCTR and could be part of putative conserved starvation and UPR modules in corals.

From the Symbiodiniaceae EVE-R analysis, we identified several divergent gene expression candidates for the lineage *Breviolum* when compared to the *Symbiodinium* A3 outgroup symbionts. *Breviolum* and *Symbiodinium sensu stricto* are thought to have diverged 147.3 MYA, whereas the *Breviolum* radiation is less than 3.4 MYA[37] (Fig. 1A). The elicited transcriptional profiles in response to heat stress was unique in *Breviolum* spp. A life history trait that may contribute to a better coupling of the symbiosis is photosymbiont mode of transmission. While *O. faveolata* and *P. clivosa* acquire Symbiodinaceae from the environment through horizontal transmission, *S. radians* acquires its photosymbiont through vertical transmission[38], leading to a stronger selective pressure imposed by the host environment.

HSP20 is an example of a gene candidate for Symbiodiniaceae expression-level adaptation (Fig. 5). HSP20 is involved in protein folding and heat stress and its expression is conserved in the two *Breviolum* species *in hospite* under control conditions. The expression in *B. faviinorum* is increased significantly in response to a temperature increase, indicating a maximum limit to the damage threshold. However, in *Breviolum* B5, expression of *HSP20* remains at the same basal level of expression even after heat treatment.

5. Intrinsic expression adaptation in response to heat stress among corals and symbiont species (extended)

Apoptosis genes have been reported in the response to heat stress in corals [9,39,40]. In our study, *BAX* (p=0.024), caspase 3 (p=0.000056), and caspase 8 (p=0.003) were found to have a divergent expression difference across conditions, which can lead to different heat susceptibilities trajectories due to the differential triggering of apoptotic transduction. *P. clivosa* expressed these transcripts more abundantly relative to *O. faveolata* and *S. radians*.

EVEReSt Symbiodinaceae divergent genes are involved in sugar transport, DNA repair, antioxidant response, lipid metabolism, photorespiration and photosynthesis. During heat stress *GLO5*, a glycolate oxidase increases slightly in expression. This enzyme, involved in photorespiration, has been proposed to act as a photoprotective mechanism during thermal

stress where it is key in diverting excessive excitation energy preventing downstream ROS production[41,42].

Along with photorespiration, all of these molecules release NH_4 which in excess is toxic to the cell. NH_4 can be assimilated via the GS-GOGAT pathway, though in plants an increase in ammonia inhibits glutamine synthase (GS)[43] promoting photorespiration during thermal stress[44]. In agreement to these observations, we found evidence for the organic nitrogen accumulation via allantoin. It has been proposed that during stress in plants, allantoins are part of a conserved mechanism to retain NH_4 in a non-toxic form[45]. An allantoin permease transcript is upregulated in *B. faviinorum* and slightly in *Breviolum* B5. Allantoins may be used as a global adaptive strategy shared among photosynthetic eukaryotes during the inhibition of GS-GOGAT cycle.

Our analysis detected a key gene to regulating the symbiont response to thermal and oxidative stress. SIR2 is a dual function protein that belongs to a family of NAD dependent deacetylases and ADP-ribosyltransferases. It acts as a central metabolic energy sensor of NAD+. Increments in this metabolite cause SIR2 increase [46-48], and in yeast[49] this increases deacetylase histones and key transcription factors involved in the antioxidant and heat shock and UPR response regulation such as catalase, SOD, and HSF1[50-52]. There is a phylogenetically conserved signal of *SIR2* expression in *Breviolum*. Both *Breviolum* B5 and *B. faviinorum* express this gene, while there is a divergent lack of expression in *Symbiodinium* A3. The *Breviolum* species have opposite photochemical efficiencies in response to a temperature increase, yet the expression of this essential key metabolic sensor/regulator is conserved.

O. faveolata can harbor multiple photosymbiont species at once from different Symbiodiniaceae genera enabling switching under elevated temperatures[53,54]. A holobiont strategy during stressful periods may rely on symbiosis disruption with a subsequent association with another photosymbiont genus. Rather than a pigment content adjustment, the strategy observed in A3 relies on the reduction of *Symbiodinium* cells. We found an expression divergent strategy mounted by the *Symbiodinium* A3 *in hospite* remaining cells. *Thioredoxin* is upregulated in those *Symbiodinium* A3 cells and not in *Breviolum* spp. Cells, highlighting an exceeding ROS production during heat stress. This protein is used to maintain an antioxidant response by reducing-oxidizing its cysteine residues redirecting the electron flow scavenging ROS.

A divergent gene involved in symbiosis maintenance expressed higher in *Breviolum* B5 but not in the other symbiont species is the α1,2 mannosyltransferase *MNN23*. In *Candida albicans*, this protein has been involved in fibrin length and in the immune recognition on the host[55]. In this yeast, the mannans resulting from cleaving are displayed on the cell walls, where they are recognized by the host innate immune system[56,57] including Toll and C-type lectins macrophage mannose receptors[58].

6. Glutathione and glyoxylate QMFs in coral holobionts

Genes involved in the glutathione biosynthesis have been found upregulated as a part of an antioxidant response during heat stress in corals[39,59] and Symbiodiniaceae[60] species. An increased glutathione metabolism QMF expression after heat stress is observed in different holobiont members in each holobiont. In the *O. faveolata* holobiont the increase occurs in the host, whereas in *S. radians* glutathione module QMF increases in its photosymbiont *Breviolum* B5 counterpart. In contrast, the associated bacterial community of *P. clivosa* increased this activity during the heat treatment (Fig. 5A). The fact that both *P. clivosa* host and its *B.*

faviinorum photosymbiont did not change glutathione expression, highlights the possibility of the thermosensitive coral species limited capacity to activate the biosynthesis of this key antioxidant during heat stress. Glyoxylate metabolism follows a consistent pattern where all the QMF holobiont members from the thermotolerant species are higher (*S. radians*) when compared to the intermediate and susceptible holobionts (*O. faveolata* and *P. clivosa*). This pathway has been described as part of corals strategy to contend with the starvation imposed by heat stress, where fatty acids are converted to carbohydrates[61] and may be key in the resistance to heat stress documented in *S. radians* holobiont thermotolerance.

7. Transcriptome sequencing, assembly statistics and annotation

Eighteen cDNA libraries, nine from coral species at the control tank and nine from corals subjected to the treatment tank were constructed and sequenced on the Illumina HiSeq 2500 platform. The number of high-quality, cleaned reads per coral species considering the three control and three treatment replicates yielded 925,234,952 total reads for *O. faveolata*, 914,039,660 total reads for *P. clivosa*, and 812,003,378 total reads for *S. radians* 150-bp, paired end cDNAs (Supplementary Fig.1; Supplementary Table 5). On average we have 114,523,717 host reads for *O. faveolata*, 80,785,437 host reads for *S. radians*, and 109,902,483 *P. clivosa* host reads. Whereas on average for the associated symbionts we have 27,576,841 *Symbiodinium* A3 reads, 57,407,871 *Breviolum* B5 reads, and 34,602,331 *Breviolum faviinorum* reads. Our high-quality transcriptomes for each coral species and associated photosymbionts are above the minimum recommended number of reads for RNA seq experiments[62].

Three distinct coral holobiont metatranscriptomes were obtained by combining these high-quality, cleaned reads of the three control and three treatment replicates per species. Each coral species was assembled separately. The total number of transcripts assembled for the *O. faveolata* holobiont was 1,297,972 (N50=1,845; largest transcript=51,329 nucleotides), whereas the *P. clivosa* holobiont result in 1,255,827 (N50=1,965; largest transcript=41,481), and although the total number of reads for *S. radians* was the lowest, the transcriptome assembly yielded the highest number of transcripts among species with a total of 2,058,347 (N50=1,405; largest transcript=41,407). Although the total number of reads was less in *S. radians,* the assembled metatranscriptome was bigger than for the other two species. The higher proportion of bacterial transcripts in *S. radians* when compared to the other species, revealed an intrinsic diverse bacterial community irrespective of the sequencing depth (Supplementary Fig.1A).

To quantify gene abundance through pseudoaligments employing kallisto[63], transcripts were prior separated per coral host, photosymbiont and bacterial associated communities by each coral species. We obtained 640,070 genes for the *O. faveolata* host, 175,772 genes from the associated *Symbiodinium* A3, and 4,928 bacterial genes. For the *P. clivosa* holobiont 428,458, 166,020, and 2,976 genes were retrieved from the host, *B. faviinorum* and bacteria respectively. *S. radians* host genes assessed were 475,058, whereas 155,089 were obtained from *Breviolum* B%, and 55,109 genes were retrieved for the associated bacterial communities inhabiting this holobiont species.

The quality of each transcriptome for the host and symbiont were evaluated by using the Benchmarking Universal Single-Copy Ortholog (BUSCO) assessment tool[64]. BUSCO analysis of the three coral hosts using a set of 954 conserved metazoan single copy orthologues as a reference, revealed *P. clivosa* host transcriptome 233 complete and single copy metazoan orthologues and 507 duplicated orthologues, resulting in 77.9% of complete orthologues. The

closely related host species, *O. faveolata* resulted in 322 complete and single copy metazoan orthologues and 395 duplicated orthologues which constitutes 75.2% complete orthologues. *S. radians* host single copy orthologues were 301 and 404 duplicated orthologues, 73.89% of complete orthologues (Supplementary Fig.1B).

Symbiodiniaceae BUSCO analysis was performed by using a set of 255 eukaryote single copy orthologues as a reference. In contrast to coral hosts completeness, symbiont transcriptomes were not as complete, which has been previously reported for other Symbiodiniaceae transcriptomes[65]. This may be reflecting a limited repertoire of genes expressed *in hospite* or a scarce representation of Symbiodiniaceae sequences in the BUSCO dataset. *Breviolum faviinorum* resulted in 108 single copy eukaryote orthologues, and 28 duplicated orthologues, 53.4% of complete orthologues. *Breviolum* B5 transcriptome yielded 62.3% of complete orthologues, from which 123 were single copy, and 36 were duplicated copy eukaryote orthologues. *Symbiodinium* A3 is the most complete transcriptome with 70.2% of complete orthologues (77 single copy, 102 duplicated copy eukaryote orthologues) (Supplementary Fig.1B).

Supplementary Figures

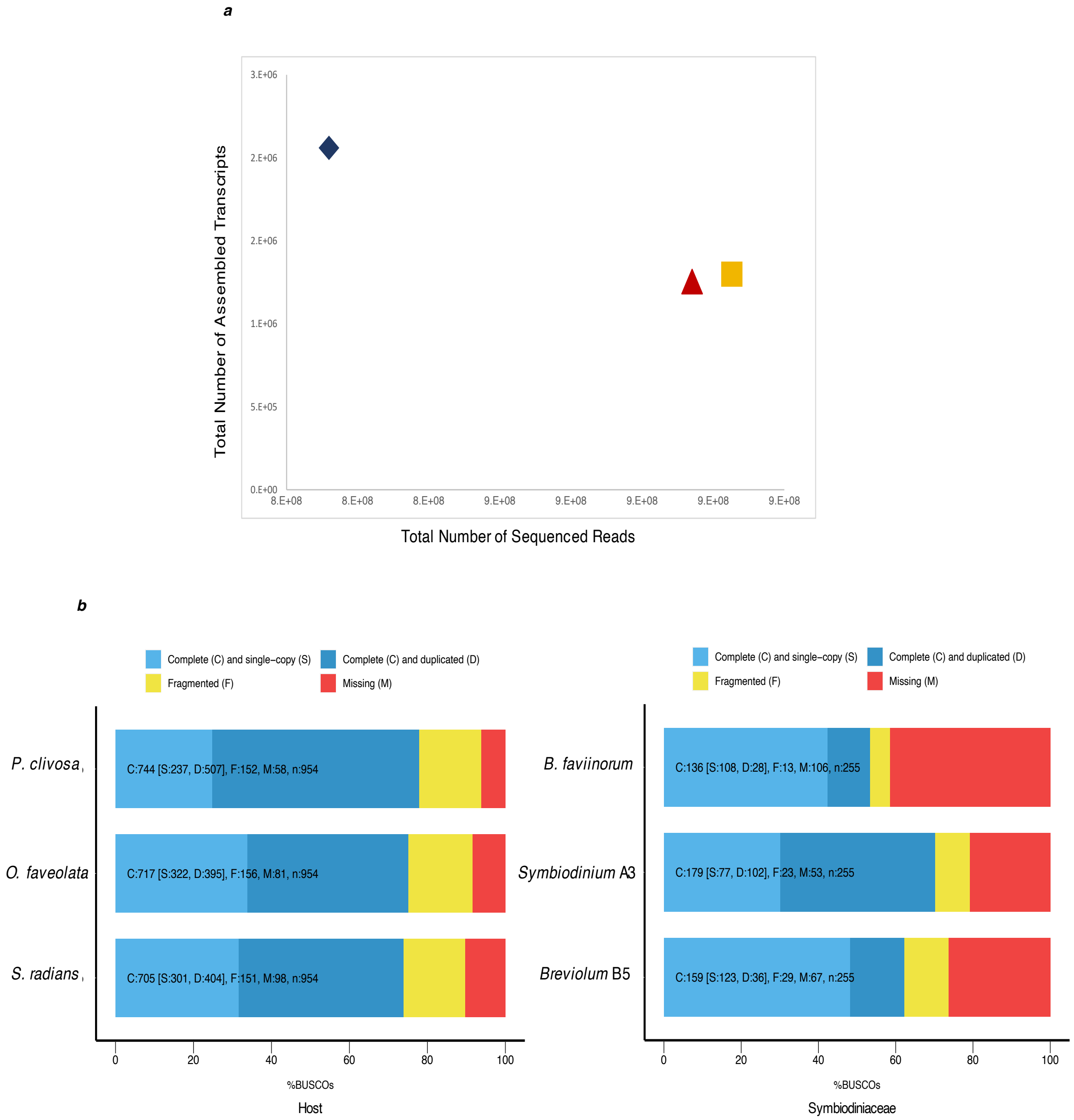

Supplementary Figure 1. Transcriptome statistics. Total number of reads *vs*. total number of assembled transcripts. The total number of reads from all the samples including control and experimental samples contributed to the metatranscriptome assemblies per coral and photosymbiont species is represented. *S. radians* (blue), *O. faveolata* (yellow), *P. clivosa* (red) (A). Cumulative percentage of orthologues inferred from the BUSCO search from the Metazoan database against the host transcriptomes from three coral species (left) and using Eukarya database against the photosymbiont transcriptomes from the three respective Symbiodiniaceae species (right) (B). Complete orthologues can be either single-copy (S) or duplicated (D); incomplete orthologues are considered fragmented (F), if orthologues from databases, they are marked as missing (M). Source data are provided as a Source Data file.

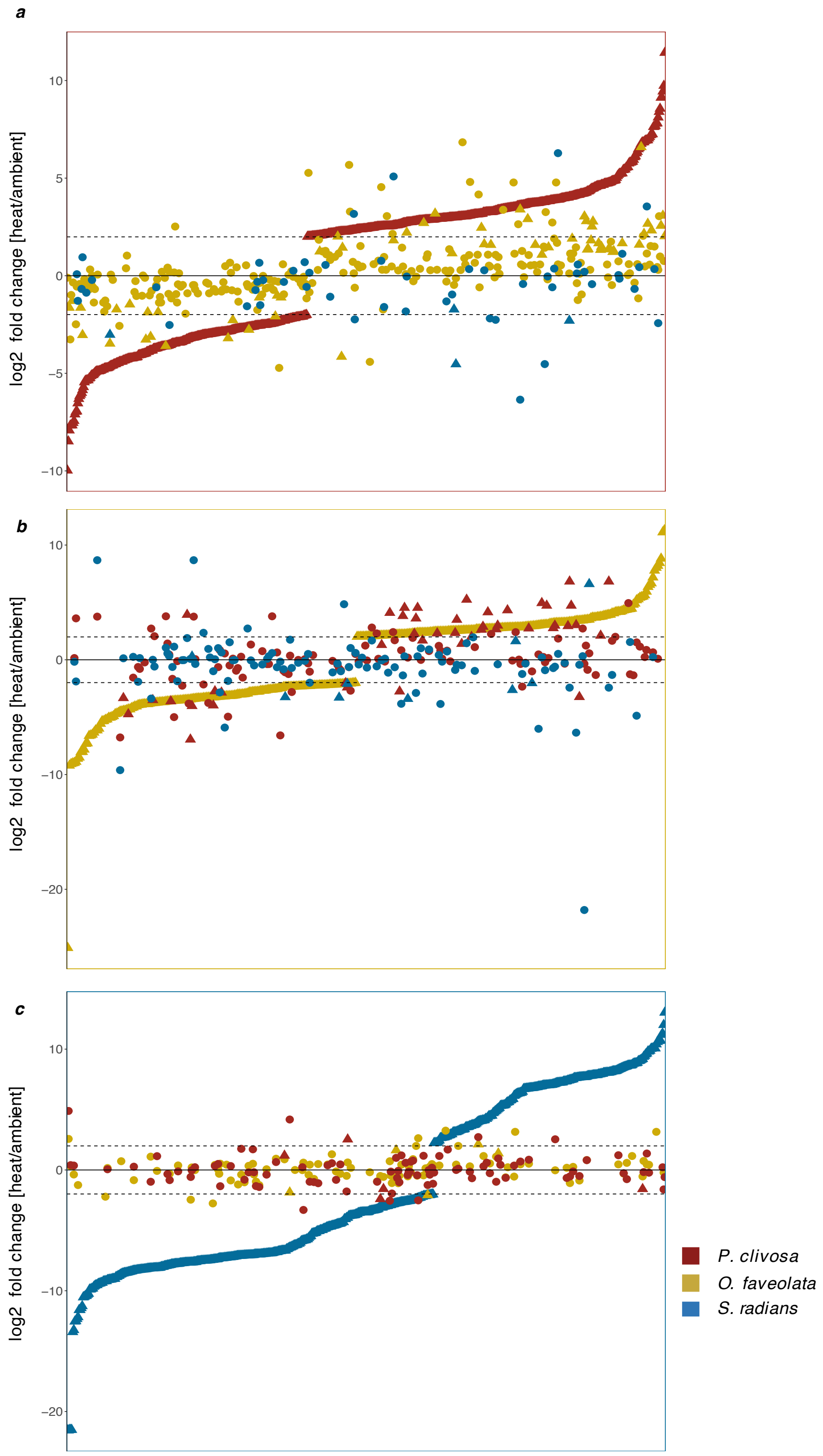

Supplementary Figure 2. Fold change of coral host ortholog expression during heat stress. DEGs using *P. clivosa* (A), *O. faveolata* (B), *S. radians* (C), as a baseline and comparing the ortholog genes across the two other coral species (-2>logFC>2, FDR<0.001). Each plot lines up DEGs according to their fold change in each species. Each plot also includes fold change for the homologues in other species. Source data are provided as a Source Data file.

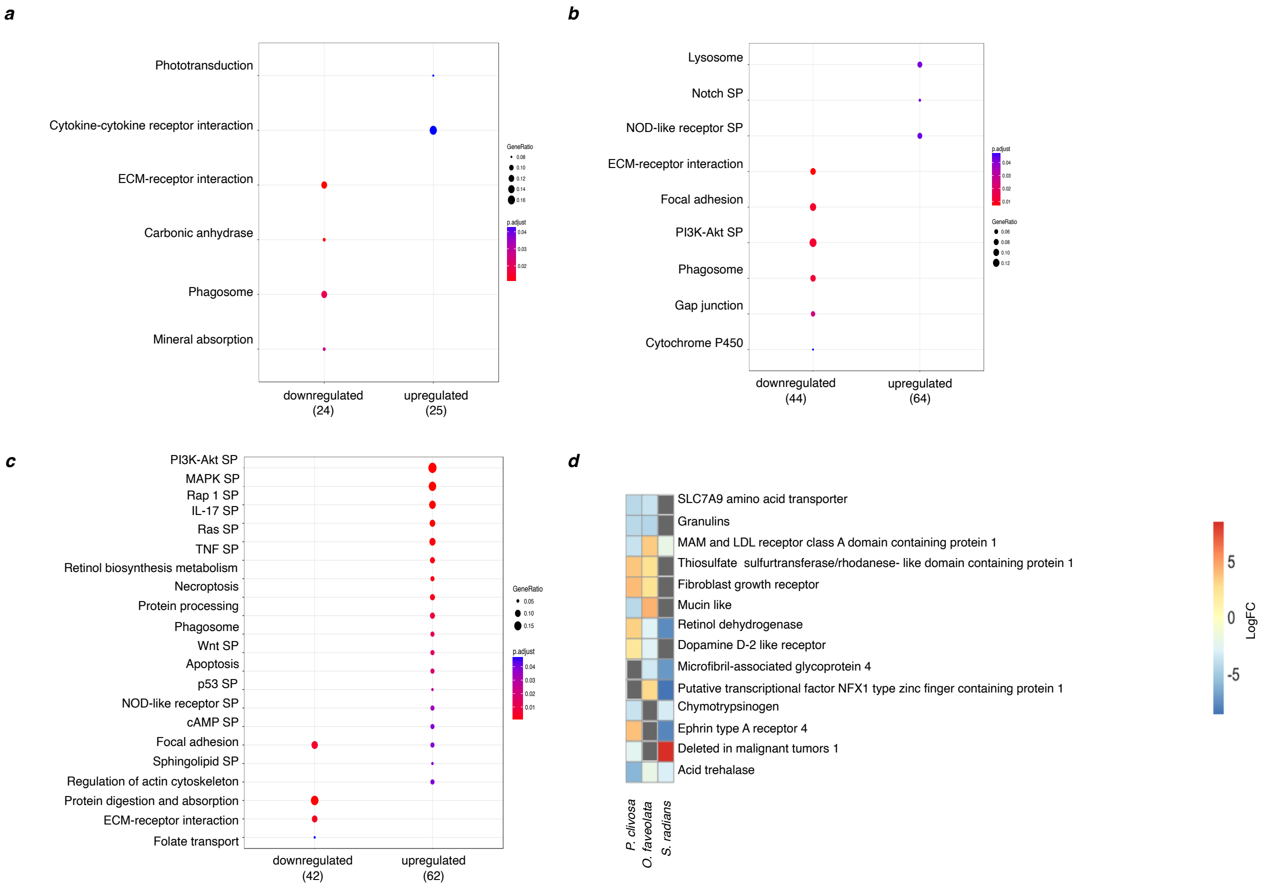

Supplementary Figure 3. Cnidarian KEGG pathway enrichment analyses on the significant DEGs after heat stress. p-value<0.05, -2>logFC>2. *S. radians* (A); *O. faveolata* (B); *P. clivosa* (C). The size of the circle is equivalent to the gene ratio and the False Discovery Rate is depicted as a heatmap. SP= Signaling pathway. (D) DEGs shared across the three coral species not described before during coral heat stress studies. The conservation of the expression profile trajectories is variable among species. Grey boxes depict genes that were not differentially expressed for a given species.

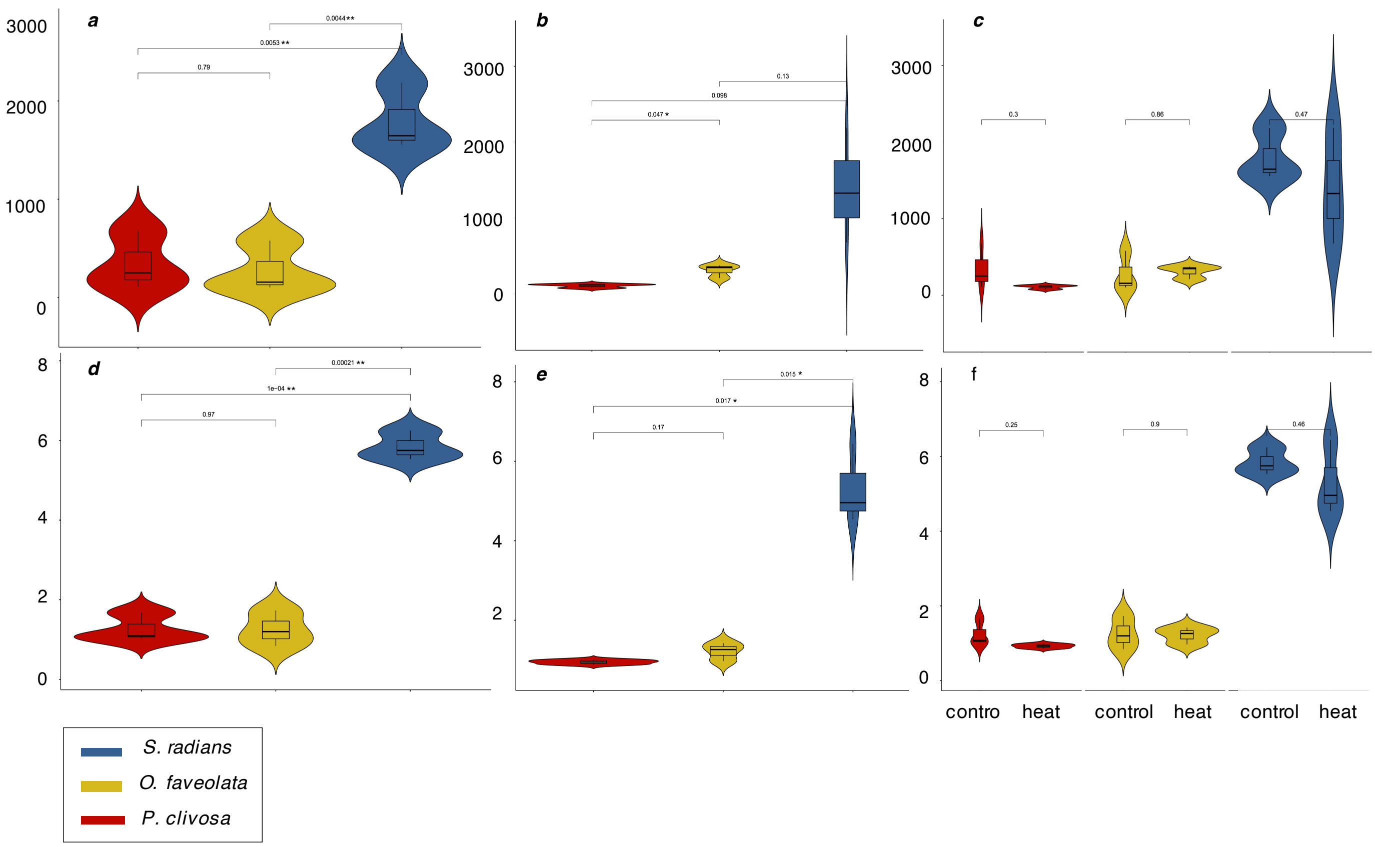

Supplementary Figure 4. Bacterial diversity and richness of the corals *S. radians, O. faveolata* and *P. clivosa*. Samples were collected after 9 days of heat treatment. A-C Chao index for bacterial richness for control fragments subject at 28ºC (A) (n=3 *S. radians*, n=3 *O. faveolata*, n=3 *P. clivosa* biologically independent coral fragments), treatment at 34ºC (B) (n=3 *S. radians*, n=3 *O. faveolata*, n=3 *P. clivosa* biologically independent coral fragments), and within species when comparing control to treatment samples (C). d-f Shannon index for diversity for control fragments subject at 28ºC (D), treatment at 34ºC (E) and within species when comparing control to treatment samples (F). Boxplot whiskers show minima and maxima; centres indicate medians; and box boundaries indicate the 25th and 75th percentiles. Two-sided t-test significance levels: ** p <0.01 and * p <0.05. Source data are provided as a Source Data file.

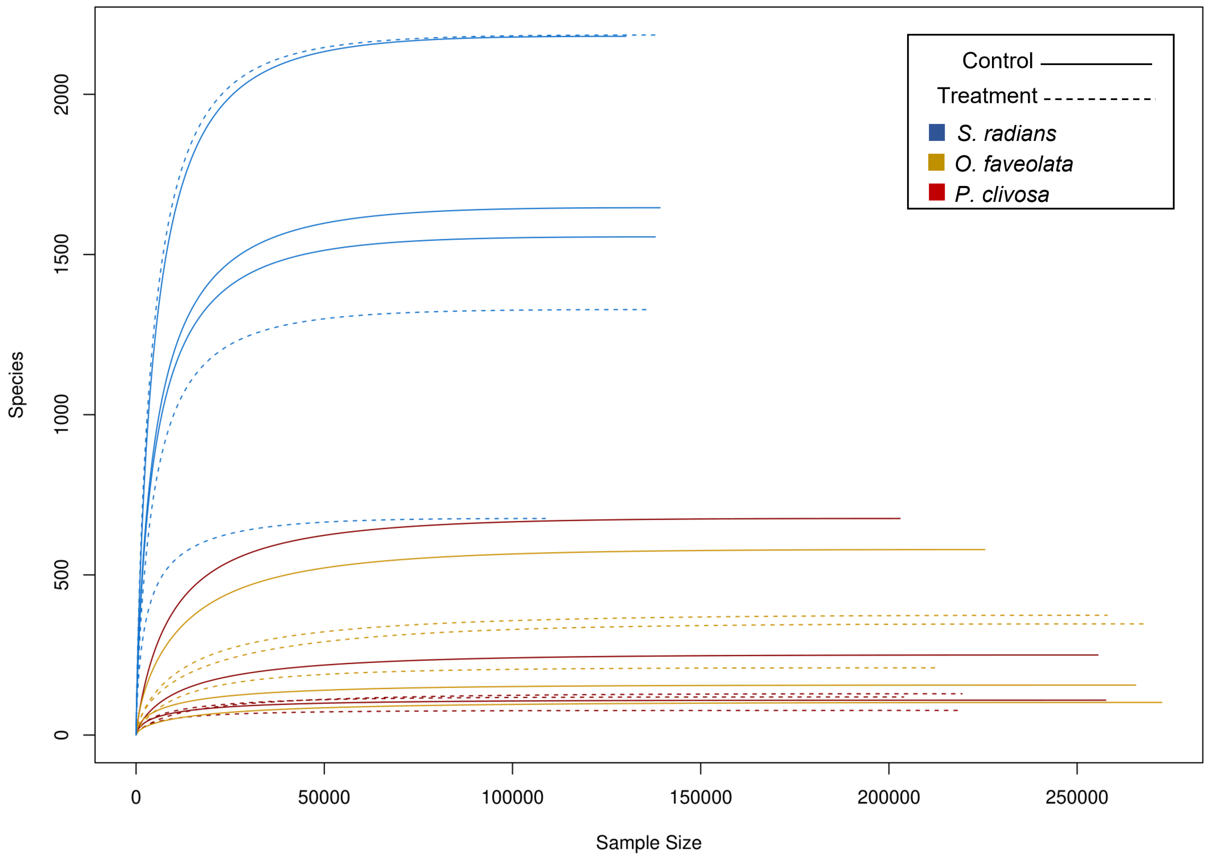

Supplementary Figure 5. Rarefaction curves for the 16S rDNA amplicon sequencing for the three coral species. Despite the differences in read count (Supplementary Table 7), the rarefaction curves for each coral species at both control (28ºC) and treatment (34ºC), reached their asymptotes consistently. This shows that the read depth was likely adequate. Source data are provided as a Source Data file.

Supplementary Tables

Supplementary Table 1. RNA Integrity for all the sequenced coral samples described in this study.

Samples	RIN	rRNA Area Ratio [28S/18S]	rRNA Height Ratio [28S/18S]
P. clivosa C A	8.4	1.779700721	1.196827049
P. clivosa C B	9.4	1.312329367	0.8108991
P. clivosa C C	9	1.340640922	0.834859427
P. clivosa T A	9	1.786400188	0.968561543
P. clivosa T B	8.4	2.312826148	1.721425475
P. clivosa T C	8.9	1.62356598	0.943475608
O. faveolata C A	8.9	1.801964339	1.045249798
O. faveolata C B	9.3	2.025412252	1.214262472
O. faveolata C C	8.9	1.765156188	1.472764766
O. faveolata T A	9.2	2.509482736	1.966165156
O. faveolata T B	9	1.856332891	1.127361976
O. faveolata T C	9.4	1.947904792	1.484973821
S. radians C A	9	1.585795103	1.322143507
S. radians C B	8.3	1.667819766	1.252786312
S. radians C C	9.4	1.735160794	1.298078034
S. radians T A	8.5	1.855798533	1.485682495
S. radians T B	9.5	1.565812748	1.120343613
S. radians T C	8.8	1.703171797	1.322320187

Supplementary Table 2. Average values ± of all parameters for the control and treatment coral fragments. Asterisks indicate differences between temperature treatments (Two-sided t-test $p<0.05$).

Parameter	At 9 days	*Breviolum* B5 (*S. radians*)	*Symbiodinium* A3 (*O. faveolata*)	*Breviolum faviinorum* (*P. clivosa*)
Symbiont density	Control 28ºC	32.84±4.78	16.35±1.06*	34.82±3.40*
	Treatment 34 ºC	35.72±5.03	6.95±0.27*	21.37±1.79*
Fv/Fm	Control 28ºC	0.65±0.008*	0.69±0.007*	0.65±0.006*
	Treatment 34 ºC	0.54±0.008*	0.45±0.02*	0.33±0.01*
a^*_{sym}	Control 28ºC	0.005±0.0007	0.016±0.0007*	0.008±0.0008
	Treatment 34 ºC	0.004±0.0004	0.027±0.002*	0.008±0.001

Supplementary Table 3. Two-sided t-test analysis comparing the physiology and optical traits of the control and treatment experiments for the three coral species investigated. Significant values (p<0.05) are indicated in bold.

Species	Parameter	df	t	p
Breviolum B5 (*S. radians*)	Symbiont density	6.9406	-0.41473	0.6909
Symbiodinium A3 (*O. faveolata*)		4.522	8.5477	**0.0005866**
Breviolum faviinorum (*P. clivosa*)		6.0639	3.4986	**0.01263**
Breviolum B5 (*S. radians*)	*Fv/Fm*	8	9.8657	**9.39E-06**
Symbiodinium A3 (*O. faveolata*)		4.5487	8.8691	**0.0004863**
Breviolum faviinorum (*P. clivosa*)		6.3296	18.952	**8.17E-07**
Breviolum B5 (*S. radians*)	a^*_{sym}	5.1445	1.4466	0.2061
Symbiodinium A3 (*O. faveolata*)		4.7321	-4.0104	**0.01142**
Breviolum faviinorum (*P. clivosa*)		7.2477	-0.52225	0.6171

Supplementary Table 4. One-way ANCOVA analyses testing species colinear relation log a^*_{symb} and log symbiont density among species. Asterisks indicate differences between symbiont species ($p<0.05$).

Parameter	Comparison		*df*	*MS*	F	*P*
Symbiont density, a^*_{symb}	*B. faviinorum* vs *Breviolum* B1	Intercept	1	0.16608	4.6452	0.0467156 *
		Slope	1	0.06436	18.0017	0.0006204 ***
	Breviolum B1 vs *Symbiodinium* A3	Intercept	1	0.30935	13.066	0.002326* *
		Slope	1	0.99371	41.972	7.615e-06***
	B. faviinorum vs *Symbiodinium* A3	Intercept	1	0.15134	5.011	0.038856*
		Slope	1	0.87188	28.868	5.061e-05***

Supplementary Table 5. The total number of paired-end reads, and the percentage of reads mapping to the host and symbiont transcriptomes and genomes. C=Control; T=treatment; ABC indicates biological replication.

Holobiont species	JGI ID	JGI alias	Total Number of Reads	Host Number of Reads	Symbiont Number of Reads
O. faveolata	1086592	8CA	151,998,400	114,441,060 (81.46%)	26,033,307 (18.53%)
	1086594	8CB	199,973,384	143,723,427 (76.64%)	43,802,082 (23.35%)
	1086596	8CC	172,942,032	119,426,161 (75.88%)	37,953,727 (24.11%)
	1086598	8TA	117,713,294	91,985,013 (86.18%)	14,753,525 (13.82%)
	1086600	8TB	128,095,550	98,552,340 (82.28%)	21,213,290 (17.71%)
	1086602	8TC	154,512,292	119,014,300 (84.57%)	21,705,112 (15.42%
S.radians	1086628	CA	121,453,656	79,257,585 (75.92%)	25,131,215 (24.07%)
	1086630	CB	140,144,298	77,164,299 (71.78%)	30,339,893 (28.22%)
	1086632	CC	147,981,344	88,741,795 (70.46%)	37,196,013 (29.53%)
	1086634	TA	69,048,362	42,610,753 (76.54%)	13,054,709 (23.45%)
	1086636	TB	184,769,196	108,150,643 (68.14%)	50,554,472 (31.85%)
	1086638	TC	148,606,522	88,787,549 (74.40%)	30,549,914 (25.60%)
P. clivosa	1086616	CA	216,699,054	147,564,875 (72%)	57,407,871 (28%)
	1086618	CB	149,435,460	96,436,820 (67.53%)	46,367,297 (32.47%)
	1086620	CC	151,618,132	103,276,784 (71.90%)	40,377,293 (28.10%)
	1086622	TA	106,937,712	80,401,691 (79.01%)	21,355,328 (20.99%)
	1086624	TB	112,539,998	90,062,010 (84.65%)	16,326,204 (15.34%)
	1086626	TC	176,809,304	141,672,719 (84.60%)	25,779,993 (15.40%)

Supplementary Table 6. Differential Expressed Genes (DEG) across coral and Symbiodiniaceae species induced by the heat stress treatment.

Member	Species	DEG Total	DEG p<0.001; -2>logFC>2	DEG p<0.01; -2>logFC>2
Host	*S. radians*	441,691	568	1,076
	O. faveolata	454,985	366	545
	P.clivosa	399,311	633	1,084
Photosymbiont	*Breviolum* B5	150,370	37	57
	Symbiodinium A3	110,593	119	216
	B. faviinorum	160,009	193	298

Supplementary Table 7. 16S rDNA amplicon sequencing total number of reads per sample yielded after QC and QIIME2 post-processing.

Holobiont species	JGI alias	Number of Reads
O. faveolata	8CA	265,563
	8CB	272,489
	8CC	225,486
	8TA	212,279
	8TB	257,984
	8TC	268,645
S. radians	CA	137,930
	CB	139,216
	CC	130,123
	TA	135,986
	TB	108,746
	TC	138,028
P. clivosa	CA	255,569
	CB	257,615
	CC	202,982
	TA	219,450
	TB	219,211
	TC	203,918

Supplementary Table 8. PCR oligonucleotide sequences used in this study.

Amplicon name	Primers name	Sequence	Reference	Notes
Symbiodiniaceae ITS2 rRNA	ITS2alg-F	5'-TCGTCGGCAGCGTCAGATGTGTATAAGAGACAGGTGAATTGCAGAACTCCGTG-3'	Pochon *et al*., 2001	rRNA gene-specific primer sequences are shown in this blue color. Illumina platform-specific sequences are shown in this green color.
	ITS2alg-R	5'-GTCTCGTGGGCTCGGAGATGTGTATAAGAGACAGCCTCCGCTTACTTATATGCTT-3'		
Archaeal and Bacterial 16S rRNA V4-V5	515F-Y	5'-GTGYCAGCMGCCGCGGTAA-3'	Parada *et al*., 2016	
	926R	5'-CCGYCAATTYMTTTRAGTTT-3'		

Supplementary Table 9. One-way ANOVA analysis comparing the optical traits of the control and treatment experiments for the three species studied. Significant values (p<0.05) are indicated in bold. Tukey HSD, P<0.05 associated with the one-way ANOVA results.

Parameter	9 days	df	MS	F	P
Symbiont density	Control 28ºC	2	501.9	10.44	**0.00287**
	Treatment 34 ºC	2	1035	21.67	**0.000104**
Fv/Fm	Control 28ºC	2	0.0019127	7.684	**0.00627**
	Treatment 34 ºC	2	0.0617	36.56	**4.59E-06**
a^*_{sym}	Control 28ºC	2	1.62E-04	57.09	**1.55E-06**
	Treatment 34 ºC	2	7.25E-04	57.36	**7.21E-07**

Tukey's HSD Comparisons

Symbiont density (C vs. T)	*Breviolum* B5 (*S. radians*)	*Symbiodinium* A3 (*O. faveolata*)
Breviolum B5 (*S. radians*)		
Symbiodinium A3 (*O. faveolata*)	0.0117092/**0.0000715**	
Breviolum faviinorum (*P. clivosa*)	0.9056419/0.0166844	**0.0038033/0.0161777**

Fv/Fm (Control/Treatment)	*Breviolum* B5 (*S. radians*)	*Symbiodinium* A3 (*O. faveolata*)
Breviolum B5 (*S. radians*)		
Symbiodinium A3 (*O. faveolata*)	0.0108222/0.0149465	
Breviolum faviinorum (*P. clivosa*)	**0.9658997/0.0000034**	**0.0129743/0.0006513**

a^*_{sym} (Control/Treatment)	*Breviolum* B5 (*S. radians*)	*Symbiodinium* A3 (*O. faveolata*)
Breviolum B5 (*S. radians*)		
Symbiodinium A3 (*O. faveolata*)	**0.0000023/0.0000009**	
Breviolum faviinorum (*P. clivosa*)	0.1222033/0.1640624	**0.0000140/0.0000088**

Supplementary References

1 Iglesias-Prieto, R., Matta, J. L., Robins, W. A. & Trench, R. K. Photosynthetic response to elevated temperature in the symbiotic dinoflagellate *Symbiodinium microadriaticum* in culture. *Proceedings of the national Academy of Sciences* **89**, 10302-10305 (1992).

2 Scheufen, T., Krämer, W. E., Iglesias-Prieto, R. & Enríquez, S. Seasonal variation modulates coral sensibility to heat-stress and explains annual changes in coral productivity. *Scientific reports* **7**, 4937 (2017).

3 Fitt, W. K., McFarland, F., Warner, M. E. & Chilcoat, G. C. Seasonal patterns of tissue biomass and densities of symbiotic dinoflagellates in reef corals and relation to coral bleaching. *Limnology and oceanography* **45**, 677-685 (2000).

4 Thornhill, D. J. *et al.* A connection between colony biomass and death in Caribbean reef-building corals. *PLoS One* **6**, e29535 (2011).

5 Oldham, M. L., Brash, A. R. & Newcomer, M. E. The structure of coral allene oxide synthase reveals a catalase adapted for metabolism of a fatty acid hydroperoxide. *Proceedings of the National Academy of Sciences* **102**, 297-302 (2005).

6 Maor-Landaw, K. & Levy, O. in *The Cnidaria, past, present and future* 523-543 (Springer, 2016).

7 Ishidate, T. *et al.* ZNFX-1 functions within perinuclear nuage to balance epigenetic signals. *Molecular cell* **70**, 639-649. e636 (2018).

8 DeSalvo, M. K., Sunagawa, S., Voolstra, C. R. & Medina, M. Transcriptomic responses to heat stress and bleaching in the elkhorn coral *Acropora palmata*. *Marine Ecology Progress Series* **402**, 97-113 (2010).

9 Barshis, D. J. *et al.* Genomic basis for coral resilience to climate change. *Proceedings of the National Academy of Sciences* **110**, 1387-1392 (2013).

10 Iturriaga, G., Suárez, R. & Nova-Franco, B. Trehalose metabolism: from osmoprotection to signaling. *International journal of molecular sciences* **10**, 3793-3810 (2009).

11 Sproles, A. E. *et al.* Phylogenetic characterization of transporter proteins in the cnidarian-dinoflagellate symbiosis. *Molecular phylogenetics and evolution* **120**, 307-320 (2018).

12 Pernice, M. *et al.* A single-cell view of ammonium assimilation in coral-dinoflagellate symbiosis. *ISME J* **6**, 1314-1324, doi:http://www.nature.com/ismej/journal/v6/n7/suppinfo/ismej2011196s1.html (2012).

13 Suescún-Bolívar, L. P., Iglesias-Prieto, R. & Thome, P. E. Induction of glycerol synthesis and release in cultured *Symbiodinium*. *PLoS One* **7**, e47182 (2012).

14 Hillyer, K. E. *et al.* Metabolite profiling of symbiont and host during thermal stress and bleaching in the coral *Acropora aspera*. *Coral Reefs* **36**, 105-118 (2017).

15 Weston, A. J. *et al.* A profile of an endosymbiont-enriched fraction of the coral *Stylophora pistillata* reveals proteins relevant to microbial-host interactions. *Molecular & Cellular Proteomics* **11**, M111. 015487 (2012).

16 Bouche, N. & Fromm, H. GABA in plants: just a metabolite? *Trends in plant science* **9**, 110-115 (2004).

17 Port, J. A. *et al.* Identification of G protein-coupled receptor signaling pathway proteins in marine diatoms using comparative genomics. *BMC genomics* **14**, 503 (2013).

18 Bullwinkle, T. *et al.* Oxidation of cellular amino acid pools leads to cytotoxic mistranslation of the genetic code. eLife, e02501. (2014).

19 DeSalvo, M., Estrada, A., Sunagawa, S. & Medina, M. Transcriptomic responses to darkness stress point to common coral bleaching mechanisms. *Coral Reefs* **31**, 215-228 (2012).
20 Sharif, R., Thomas, P., Zalewski, P. & Fenech, M. The role of zinc in genomic stability. *Mutation Research/Fundamental and Molecular Mechanisms of Mutagenesis* **733**, 111-121 (2012).
21 Liuzzi, J. P., Guo, L., Yoo, C. & Stewart, T. S. Zinc and autophagy. *Biometals* **27**, 1087-1096 (2014).
22 Oteiza, P. I. Zinc and the modulation of redox homeostasis. *Free Radical Biology and Medicine* **53**, 1748-1759 (2012).
23 Harland, A., Bryan, G. & Brown, B. Zinc and cadmium absorption in the symbiotic anemone *Anemonia viridis* and the non-symbiotic anemone Actinia equina. *Journal of the Marine Biological Association of the United Kingdom* **70**, 789-802 (1990).
24 Lehnert, E. M. *et al.* Extensive differences in gene expression between symbiotic and aposymbiotic cnidarians. *G3: Genes, Genomes, Genetics* **4**, 277-295 (2014).
25 Erdner, D. L. & Anderson, D. M. Ferredoxin and flavodoxin as biochemical indicators of iron limitation during open-ocean iron enrichment. *Limnology and oceanography* **44**, 1609-1615 (1999).
26 Frederick, J. R. & Petri, W. A., Jr. Roles for the galactose-/N-acetylgalactosamine-binding lectin of *Entamoeba* in parasite virulence and differentiation. *Glycobiology* **15**, 53R-59R, doi:10.1093/glycob/cwj007 (2005).
27 Lundgren, P., Vera, J. C., Peplow, L., Manel, S. & van Oppen, M. J. Genotype - environment correlations in corals from the Great Barrier Reef. *BMC Genet* **14**, 9, doi:10.1186/1471-2156-14-9 (2013).
28 Holt, J. F. *et al. Enterococcus faecalis* 6-phosphogluconolactonase is required for both commensal and pathogenic interactions with *Manduca sexta*. *Infect Immun* **83**, 396-404, doi:10.1128/IAI.02442-14 (2015).
29 McGinty, E. S., Pieczonka, J. & Mydlarz, L. D. Variations in reactive oxygen release and antioxidant activity in multiple *Symbiodinium* types in response to elevated temperature. *Microb Ecol* **64**, 1000-1007, doi:10.1007/s00248-012-0085-z (2012).
30 Oakley, C. A. *et al.* Thermal shock induces host proteostasis disruption and endoplasmic reticulum stress in the model symbiotic cnidarian *Aiptasia*. *Journal of proteome research* **16**, 2121-2134 (2017).
31 Levy, O. *et al.* Molecular assessment of the effect of light and heterotrophy in the scleractinian coral *Stylophora pistillata*. *Proceedings of the Royal Society B: Biological Sciences* **283**, 20153025 (2016).
32 Tao, J., Ma, Y.-C., Yang, Z.-S., Zou, C.-G. & Zhang, K.-Q. Octopamine connects nutrient cues to lipid metabolism upon nutrient deprivation. *Science advances* **2**, e1501372 (2016).
33 Aonuma, H. *et al.* Weak involvement of octopamine in aversive taste learning in a snail. *Neurobiology of learning and memory* **141**, 189-198 (2017).
34 Chan, C.-P., Kok, K.-H. & Jin, D.-Y. CREB3 subfamily transcription factors are not created equal: Recent insights from global analyses and animal models. *Cell & bioscience* **1**, 6 (2011).
35 Bravo, R. *et al.* in *International review of cell and molecular biology* Vol. 301 215-290 (Elsevier, 2013).
36 Saito, A. *et al.* Regulation of endoplasmic reticulum stress response by a BBF2H7-mediated Sec23a pathway is essential for chondrogenesis. *Nature cell biology* **11**, 1197 (2009).

37 LaJeunesse, T. C. *et al.* Systematic revision of Symbiodiniaceae highlights the antiquity and diversity of coral endosymbionts. *Current Biology* **28**, 2570-2580. e2576 (2018).
38 Harrison, P. L. & Wallace, C. Reproduction, dispersal and recruitment of scleractinian corals. *Ecosystems of the world* **25**, 133-207 (1990).
39 DeSalvo, M. *et al.* Differential gene expression during thermal stress and bleaching in the Caribbean coral *Montastraea faveolata*. *Molecular ecology* **17**, 3952-3971 (2008).
40 Bay, R. A. & Palumbi, S. R. Multilocus adaptation associated with heat resistance in reef-building corals. *Current Biology* **24**, 2952-2956 (2014).
41 Smith, D. J., Suggett, D. J. & Baker, N. R. Is photoinhibition of zooxanthellae photosynthesis the primary cause of thermal bleaching in corals? *Global Change Biology* **11**, 1-11 (2005).
42 Foyer, C. H., Bloom, A. J., Queval, G. & Noctor, G. Photorespiratory metabolism: genes, mutants, energetics, and redox signaling. *Annual review of plant biology* **60**, 455-484 (2009).
43 Mattsson, M. & Schjoerring, J. K. Ammonia emission from young barley plants: influence of N source, light/dark cycles and inhibition of glutamine synthetase. *Journal of Experimental Botany* **47**, 477-484 (1996).
44 Mattsson, M., Hausler, R. E., Leegood, R. C., Lea, P. J. & Schjoerring, J. K. Leaf-atmosphere NH3 exchange in barley mutants with reduced activities of glutamine synthetase. *Plant Physiology* **114**, 1307-1312 (1997).
45 Casartelli, A. *et al.* Opposite fates of the purine metabolite allantoin under water and nitrogen limitations in bread wheat. *Plant molecular biology*, 1-21 (2019).
46 Ahn, B.-H. *et al.* A role for the mitochondrial deacetylase Sirt3 in regulating energy homeostasis. *Proceedings of the National Academy of Sciences* **105**, 14447-14452 (2008).
47 Gambini, J. *et al.* Free [NADH]/[NAD+] regulates sirtuin expression. *Archives of biochemistry and biophysics* **512**, 24-29 (2011).
48 Osborne, B., Cooney, G. J. & Turner, N. Are sirtuin deacylase enzymes important modulators of mitochondrial energy metabolism? *Biochimica et Biophysica Acta (BBA)-General Subjects* **1840**, 1295-1302 (2014).
49 Imai, S.-I., Armstrong, C. M., Kaeberlein, M. & Guarente, L. Transcriptional silencing and longevity protein Sir2 is an NAD-dependent histone deacetylase. *Nature* **403**, 795 (2000).
50 Wang, F., Nguyen, M., Qin, F. X. F. & Tong, Q. SIRT2 deacetylates FOXO3a in response to oxidative stress and caloric restriction. *Aging cell* **6**, 505-514 (2007).
51 Weindling, E. & Bar-Nun, S. Sir2 links the unfolded protein response and the heat shock response in a stress response network. *Biochemical and biophysical research communications* **457**, 473-478 (2015).
52 Westerheide, S. D., Anckar, J., Stevens, S. M., Sistonen, L. & Morimoto, R. I. Stress-inducible regulation of heat shock factor 1 by the deacetylase SIRT1. *Science* **323**, 1063-1066 (2009).
53 Pettay, D. T., Wham, D. C., Smith, R. T., Iglesias-Prieto, R. & LaJeunesse, T. C. Microbial invasion of the Caribbean by an Indo-Pacific coral zooxanthella. *Proceedings of the National Academy of Sciences* **112**, 7513-7518 (2015).
54 Kemp, D. W., Hernandez-Pech, X., Iglesias-Prieto, R., Fitt, W. K. & Schmidt, G. W. Community dynamics and physiology of *Symbiodinium* spp. before, during, and after a coral bleaching event. *Limnology and Oceanography* **59**, 788-797 (2014).
55 Hall, R. A. *et al.* The Mnn2 mannosyltransferase family modulates mannoprotein fibril length, immune recognition and virulence of *Candida albicans*. *PLoS pathogens* **9**, e1003276 (2013).

56 Netea, M. G., Brown, G. D., Kullberg, B. J. & Gow, N. A. An integrated model of the recognition of Candida albicans by the innate immune system. *Nature Reviews Microbiology* **6**, 67 (2008).
57 Brown, G. D. & Gordon, S. Immune recognition: a new receptor for β-glucans. *Nature* **413**, 36 (2001).
58 Netea, M. G. *et al.* Variable recognition of *Candida albicans* strains by TLR4 and lectin recognition receptors. *Medical mycology* **48**, 897-903 (2010).
59 Kirk, N. L., Howells, E. J., Abrego, D., Burt, J. A. & Meyer, E. Genomic and transcriptomic signals of thermal tolerance in heat-tolerant corals (*Platygyra daedalea*) of the Arabian/Persian Gulf. *Molecular ecology* **27**, 5180-5194 (2018).
60 Krueger, T. *et al.* Antioxidant plasticity and thermal sensitivity in four types of *Symbiodinium* sp. *Journal of phycology* **50**, 1035-1047 (2014).
61 Kenkel, C. D. & Matz, M. V. Gene expression plasticity as a mechanism of coral adaptation to a variable environment. *Nature Ecology & Evolution* **1**, 1-6 (2016).
62 Baccarella, A., Williams, C. R., Parrish, J. Z. & Kim, C. C. Empirical assessment of the impact of sample number and read depth on RNA-Seq analysis workflow performance. *BMC bioinformatics* **19**, 423 (2018).
63 Bray, N. L., Pimentel, H., Melsted, P. & Pachter, L. Near-optimal probabilistic RNA-seq quantification. *Nature biotechnology* **34**, 525-527 (2016).
64 Seppey, M., Manni, M. & Zdobnov, E. M. in *Gene Prediction* 227-245 (Springer, 2019).
65 Shah, S., Chen, Y., Bhattacharya, D. & Chan, C. X. Sex in Symbiodiniaceae dinoflagellates: genomic evidence for independent loss of the canonical synaptonemal complex. *Scientific reports* **10**, 1-12 (2020).
66 Louis, Y. D., Bhagooli, R., Kenkel, C. D., Baker, A. C. & Dyall, S. D. Gene expression biomarkers of heat stress in scleractinian corals: promises and limitations. *Comparative Biochemistry and Physiology Part C: Toxicology & Pharmacology* **191**, 63-77 (2017).
