# Supplementary material for: Elucidating gene expression adaptation of phylogenetically divergent coral holobionts under heat stress
Source: Nat Commun. 2021 Sep 30;12:5731. doi: 10.1038/s41467-021-25950-4 (PMC8484447; doi:10.1038/s41467-021-25950-4)
Supplement: Supplementary file 2 — Description of Additional Supplementary Files [file 41467_2021_25950_MOESM2_ESM.pdf]

Description of Additional Supplementary Files

Title: Supplementary Data 1.
Description: Hosts and associated photosymbionts differential expression analyses.

Title: Supplementary Data 2.
Description: Host and associated photosymbionts EVE analyses (EVE-R and EVEReSt).

Title: Supplementary Data 3.
Description: Host and associated photosymbionts GO MWU analyses.
