# Supplementary material for: Elucidating gene expression adaptation of phylogenetically divergent coral holobionts under heat stress
Source: Nat Commun. 2021 Sep 30;12:5731. doi: 10.1038/s41467-021-25950-4 (PMC8484447; doi:10.1038/s41467-021-25950-4)
Supplement: Supplementary file 6 — Reporting Summary [file 41467_2021_25950_MOESM6_ESM.pdf]

nature research

Corresponding author(s): Viridiana Avila-Magaña, Mónica Medina

Last updated by author(s): August 9th, 2021

Reporting Summary

Nature Research wishes to improve the reproducibility of the work that we publish. This form provides structure for consistency and transparency in reporting. For further information on Nature Research policies, see our Editorial Policies and the Editorial Policy Checklist.

Statistics

For all statistical analyses, confirm that the following items are present in the figure legend, table legend, main text, or Methods section.

n/a	Confirmed	
[ ]	[x]	The exact sample size (*n*) for each experimental group/condition, given as a discrete number and unit of measurement
[ ]	[x]	A statement on whether measurements were taken from distinct samples or whether the same sample was measured repeatedly
[ ]	[x]	The statistical test(s) used AND whether they are one- or two-sided<br>*Only common tests should be described solely by name; describe more complex techniques in the Methods section.*
[ ]	[x]	A description of all covariates tested
[ ]	[x]	A description of any assumptions or corrections, such as tests of normality and adjustment for multiple comparisons
[ ]	[x]	A full description of the statistical parameters including central tendency (e.g. means) or other basic estimates (e.g. regression coefficient) AND variation (e.g. standard deviation) or associated estimates of uncertainty (e.g. confidence intervals)
[ ]	[x]	For null hypothesis testing, the test statistic (e.g. *F*, *t*, *r*) with confidence intervals, effect sizes, degrees of freedom and *P* value noted<br>*Give P values as exact values whenever suitable.*
[x]	[ ]	For Bayesian analysis, information on the choice of priors and Markov chain Monte Carlo settings
[x]	[ ]	For hierarchical and complex designs, identification of the appropriate level for tests and full reporting of outcomes
[x]	[ ]	Estimates of effect sizes (e.g. Cohen's *d*, Pearson's *r*), indicating how they were calculated

Our web collection on statistics for biologists contains articles on many of the points above.

Software and code

Policy information about availability of computer code

Data collection	No software was used. N/A.
Data analysis	Bbduk(v35.x), BBmap(v35.x), Trinity (version 2.4.0), Kallisto (version 0.43.0), DeSeq2 (as part of Trinity DE pipeline, R package(v.3.5.1)), OrthoFinder (version 2.4.0), BLAST 2.2.31, diamond (version 0.8.22.84), MEGAN5, FastQC, USEARCH (version 6.1), FLASH (version 1.2.11), QIIME2 (v2017.7), microbiome R package (v. 1.13.7), EVE(v.0), Agalma (version 0.5.0), RBGOA (https://github.com/z0on/GO_MWU), vegan R package (v2.5-6), phyloseq R package (v1.32.0), eggNOG-mapper(v2.0), BUSCO (v4.1.4). The code to replicate our analyses is available on dryad repository (https://doi.org/10.5061/dryad.k3j9kd57b).

For manuscripts utilizing custom algorithms or software that are central to the research but not yet described in published literature, software must be made available to editors and reviewers. We strongly encourage code deposition in a community repository (e.g. GitHub). See the Nature Research guidelines for submitting code & software for further information.

Data

Policy information about availability of data

All manuscripts must include a data availability statement. This statement should provide the following information, where applicable:

- Accession codes, unique identifiers, or web links for publicly available datasets
- A list of figures that have associated raw data
- A description of any restrictions on data availability

All raw sequencing data that support the findings of this study have been deposited in the National Center for Biotechnology Information Sequencing Reading Archive (SRA) and are accessible through the SRA Series accession numbers: “SRP123687 [https://www.ncbi.nlm.nih.gov/sra/?term=SRP123687]”, “SRP123688 [https://www.ncbi.nlm.nih.gov/sra/?term=SRP123688]”, “SRP123689 [https://www.ncbi.nlm.nih.gov/sra/?term=SRP123689]”, “SRP123701 [https://www.ncbi.nlm.nih.gov/sra/?term=SRP123701]”, “SRP123703 [https://www.ncbi.nlm.nih.gov/sra/?term=SRP123703]”, “SRP123705 [https://www.ncbi.nlm.nih.gov/sra/?term=SRP123705]”, “SRP123711 [https://www.ncbi.nlm.nih.gov/sra/?term=SRP123711]”, “SRP123713 [https://www.ncbi.nlm.nih.gov/sra/?term=SRP123713]”, “SRP123717 [https://www.ncbi.nlm.nih.gov/sra/?term=SRP123717]”, “SRP123716 [https://www.ncbi.nlm.nih.gov/sra/?term=SRP123716]”, “SRP123715 [https://www.ncbi.nlm.nih.gov/sra/?term=SRP123715]”, “SRP123719 [https://www.ncbi.nlm.nih.gov/sra/?term=SRP123719]”, “SRP123718 [https://www.ncbi.nlm.nih.gov/sra/?term=SRP123718]”, “SRP123722 [https://www.ncbi.nlm.nih.gov/sra/?term=SRP123722]”, “SRP123725 [https://www.ncbi.nlm.nih.gov/sra/?term=SRP123725]”, “SRP123724 [https://www.ncbi.nlm.nih.gov/sra/?term=SRP123724]”, “SRP123726 [https://www.ncbi.nlm.nih.gov/sra/?term=SRP123726]”, “SRP123727 [https://www.ncbi.nlm.nih.gov/sra/?term=SRP123727]”, “SRP225190 [https://www.ncbi.nlm.nih.gov/sra/? [illegible]

Field-specific reporting

Please select the one below that is the best fit for your research. If you are not sure, read the appropriate sections before making your selection.

☐ Life sciences ☐ Behavioural & social sciences ☒ Ecological, evolutionary & environmental sciences

For a reference copy of the document with all sections, see nature.com/documents/nr-reporting-summary-flat.pdf

Ecological, evolutionary & environmental sciences study design

All studies must disclose on these points even when the disclosure is negative.

Study description

We collected a total of 10 biological replicates from different coral colonies of three Caribbean corals: Siderastrea radians, Orbicella faveolata and Pseudodiploria clivosa. S. radians samples consisted of small juvenile colonies. Coral fragments of O. faveolata and P. clivosa were obtained by using a 2 cm in diameter coring device. Following the 16 days of acclimation, heaters were turned on for one of the tanks. Temperature in the control tank remained at ~28ºC, while in the heat-treatment tank was increased to 32ºC ± 0.34 at day 0.Photosynthetically active radiation (PAR) measured at noon was average 230 ± 57 mol quanta m-2 s-1. As no signs of changes in coral color were observed for 7 days, water temperature was increased to 34ºC ± 0.30 and maintained for 2 days until the end of the experiment. The stress period lasted a total of nine days.

Research sample

We collected a total of 10 biological replicates from different coral colonies of three Caribbean coral species: Siderastrea radians, Orbicella faveolata and Pseudodiploria clivosa. S. radians samples consisted of small juvenile unattached colonies around the same size taken from a sea grass bed. Coral fragments of O. faveolata and P. clivosa were obtained by chiseling a 5 cm x 5 cm piece of the coral colony with a metal coring device of 2 cm in diameter. These samples were chosen randomly to represent the respective populations of the studied species.

Sampling strategy

Five colonies of each species were randomly selected at near the Universidad Nacional Autonoma de Mexico's Coral Reef Unit in Puerto Morelos, Quintana Roo, in November 2008. Due to a prior massive bleaching event, we decided to collect conservatively in order to reduce impact to the reef. We were also limited by the number of specimens allowed in the collection permit. At that time, previous transcriptomic studies conducted in our lab and other labs, using the same number of replicates, shed light on the physiological and molecular basis of coral bleaching in several Caribbean coral species.

Data collection

Photophysiology parameters (i.e., photochemical efficiency, photosymbiont cell density, and cell reflectance) were measured and collected by Michael DeSalvo at Instituto de Ciencias del Mar y Limnologia, UNAM, Puerto Morelos. The data was recorded both in a field trip notebook and electronically in Microsoft Excel datasheets.

Timing and spatial scale

All samples were collected at the same time after 9 days of heat exposure in experimental tanks (a control tank at 28C and a treatment tank at 34C) during November 2008. The sampling time was based on the photophysiology measurements and previous studies conducted by us and other published data.

Data exclusions

No data was excluded.

Reproducibility

The experiment was conducted during Winter, and although there maybe a seasonal variation, all the species were subjected to the same experimental conditions to reduce environmental variability. Due to challenges intrinsic to physiological experiments, the characterization of the physiological response is usually constrained to a small number of replicates. Previous to the experiment, the system was characterized to minimize spatial variability in irradiance, water flow conditions, and temporal changes in seawater temperature within each tank. In addition to this, measurements of photochemical efficiency allow us to recognize fast any significant not-controlled source of variability during the experiment that could alter the physiological condition of the samples at any time or in any particular section of the tank. We aimed to induce heat stress with very contrasting temperature regimes and under similar and fully controlled external light environments. Previous studies focusing in coral photo-physiology have shown a characteristic response to heat stress by the three target species in our study. We are thus confident in the reproducibility of our study. We did not replicate this study outside of that specific field season.

Randomization

Randomization is not relevant to this particular experimental setup, as all the replicates were placed with the same controlled irradiance, water flow conditions, within each tank to minimize the tank effect and variability.

Blinding

Not applicable, the RNA was sequenced at the Joint Genome Institute, those conducting the sequencing were unaware of the experimental setup as the samples received unique IDs prior to sequencing that are unrelated to the experimental design.

Did the study involve field work? ☒ Yes ☐ No

Field work, collection and transport

Field conditions

In order to control the environmental parameters at the field conditions we performed a tank experiment where we controlled the temperature (26° C at control tank and 34° C at heat treatment tank). Corals were collected in Winter 2008.

Location

Orbicella faveolata and Pseudodiploria clivosa coral fragments were collected at La Bocana, Puerto Morelos, Mexico (20° 52' 40" N, 86° 50' 35" W) at a 4-7 meters depth, whereas colonies from Siderastrea radians were collected in El Islote, Puerto Morelos, Mexico (20°55.607’N, 86°49.882’W) at 2 meters depth.

Access & import/export

Coral samples were collected in Puerto Morelos, Mexico during November 2008 on the permit registration MX-HR-010-MEX folio 016.

Disturbance	Care was taken to not disturb the reef, and all sampling complied with the local collection guidelines.

Reporting for specific materials, systems and methods

We require information from authors about some types of materials, experimental systems and methods used in many studies. Here, indicate whether each material, system or method listed is relevant to your study. If you are not sure if a list item applies to your research, read the appropriate section before selecting a response.

Materials & experimental systems

n/a	Involved in the study
☒	☐ Antibodies
☒	☐ Eukaryotic cell lines
☒	☐ Palaeontology and archaeology
☐	☒ Animals and other organisms
☒	☐ Human research participants
☒	☐ Clinical data
☒	☐ Dual use research of concern

Methods

n/a	Involved in the study
☒	☐ ChIP-seq
☒	☐ Flow cytometry
☒	☐ MRI-based neuroimaging

Animals and other organisms

Policy information about studies involving animals; ARRIVE guidelines recommended for reporting animal research

Laboratory animals	The study did not involve laboratory animals
Wild animals	Coral fragments from three species: Orbicella faveolata, Siderastrea radians, and Pseudodiploria clivosa were collected in the field, and kept at the tanks for a 16 days acclimation period at the Instituto de Ciencias del Mar y Limonologia, UNAM. Control fragments were kept at 28ºC, whereas heat treated corals were kept at 32ºC for 7 days, as no signs of changes in coral color were observed for 7 days, water temperature was increased to 34ºC. After 2 days, coral fragments, both from control and treatment tanks (experiment total duration was 9 days) were wrapped in foiled flash frozen with Liquid Nitrogen and transported in a dry shipper to United States.
Field-collected samples	Following the 16 days of acclimation, heaters were turned on for one of the tanks. Temperature in the control tank remained at ~28ºC, while in the heat-treatment tank was increased to 32ºC ± 0.34 at day 0. Photosynthetically active radiation (PAR) measured at noon was average 230 ± 57 µmol quanta m-2 s-1. As no signs of changes in coral color were observed for 7 days, water temperature was increased to 34ºC ± 0.30 and maintained for 2 days until the end of the experiment.
Ethics oversight	Animals/Experiments used in this study do not required ethical approval. However, we did include the collection permit number which corresponds to the local authorities' number as well as CITES.: Sample collection and export number MX-HR-010-MEX folio 016.

Note that full information on the approval of the study protocol must also be provided in the manuscript.
